# Monosynaptic Rabies Tracing Reveals Sex- and Age-Dependent Dorsal Subiculum Connectivity Alterations in an Alzheimer's Disease Mouse Model

**DOI:** 10.1523/JNEUROSCI.1796-23.2024

**Published:** 2024-03-19

**Authors:** Qiao Ye, Gocylen Gast, Erik George Wilfley, Hanh Huynh, Chelsea Hays, Todd C. Holmes, Xiangmin Xu

**Affiliations:** ^1^Department of Anatomy and Neurobiology, School of Medicine, University of California, Irvine, California 92697; ^2^Department of Biomedical Engineering, University of California, Irvine, California 92697; ^3^Department of Physiology and Biophysics, School of Medicine, University of California, Irvine, California 92697; ^4^Center for Neural Circuit Mapping, University of California, Irvine, California 92697

**Keywords:** Alzheimer's disease, monosynaptic, neural circuit, rabies, retrograde, subiculum

## Abstract

The subiculum (SUB), a hippocampal formation structure, is among the earliest brain regions impacted in Alzheimer's disease (AD). Toward a better understanding of AD circuit-based mechanisms, we mapped synaptic circuit inputs to dorsal SUB using monosynaptic rabies tracing in the 5xFAD mouse model by quantitatively comparing the circuit connectivity of SUB excitatory neurons in age-matched controls and 5xFAD mice at different ages for both sexes. Input-mapped brain regions include the hippocampal subregions (CA1, CA2, CA3), medial septum and diagonal band, retrosplenial cortex, SUB, postsubiculum (postSUB), visual cortex, auditory cortex, somatosensory cortex, entorhinal cortex, thalamus, perirhinal cortex (Prh), ectorhinal cortex, and temporal association cortex. We find sex- and age-dependent changes in connectivity strengths and patterns of SUB presynaptic inputs from hippocampal subregions and other brain regions in 5xFAD mice compared with control mice. Significant sex differences for SUB inputs are found in 5xFAD mice for CA1, CA2, CA3, postSUB, Prh, lateral entorhinal cortex, and medial entorhinal cortex: all of these areas are critical for learning and memory. Notably, we find significant changes at different ages for visual cortical inputs to SUB. While the visual function is not ordinarily considered defective in AD, these specific connectivity changes reflect that altered visual circuitry contributes to learning and memory deficits. Our work provides new insights into SUB-directed neural circuit mechanisms during AD progression and supports the idea that neural circuit disruptions are a prominent feature of AD.

## Significance Statement

Alzheimer's disease (AD) is a major health concern in the elderly, but the underlying neural circuit mechanisms of this disease remain unclear. The subiculum (SUB) is a critical brain region for relaying and integrating hippocampal and cortical information. In this study, we applied monosynaptic rabies viral tracing to study the circuit connectivity changes of SUB excitatory neurons in age-matched, gender-balanced control and 5xFAD mice. We identified age-progressive alterations of connectivity strengths and patterns of SUB neurons in AD model mice. The circuit alterations are differentially impacted in different genders for specific brain regions. Our new findings are supported by human AD literature and can help to identify potential new therapeutic circuit targets for AD treatments.

## Introduction

Alzheimer's disease (AD) is the most common form of dementia and a major health concern worldwide. AD age-progressive cognitive decline is correlated with extensive synaptic and neuronal loss. However, the temporal relationship between these behavioral and brain structural connectivity changes remains poorly understood. Recent evidence has suggested that disruption of long-range and local neural circuit connections is a functionally critical defect in human AD patients and animal models (Busche et al., 2015; Jeon et al., 2018; Harris et al., 2020). DiI-based retrograde neural circuit tracing of the hippocampus in the 5xFAD mouse model demonstrates decreased hippocampal inputs from the medial septum and diagonal band (MS-DB), entorhinal cortex (EC), auditory (Aud) cortex, locus ceruleus, dorsal raphe, substantia nigra pars compacta, and olfactory bulb (Jeon et al., 2018). Our recent work using monosynaptic rabies virus tracing reveals substantial connectivity alterations of the hippocampal CA1 excitatory neurons in the amyloid precursor protein knock-in (APP-KI) mouse model (Ye et al., 2022). Furthermore, AD prevalence differs between males and females, but the underlying basis of this remains poorly understood. In humans, females have a higher risk of developing pathological features of AD than men who are of the same age (Seshadri et al., 1997; Calvo and Einstein, 2023). We recently have found sex differences in CA1 connectivity and patterns in APP-KI mice (Ye et al., 2022). Specifically, aged AD female mice have significantly lower SUB to CA1 connectivity defects compared with aged AD male mice.

Our present work focuses on the subiculum (SUB), a brain region that plays a role in spatial navigation, mnemonic processing, and control of the stress response (O'Mara, 2005). The degeneration of vulnerable pyramidal neurons in the SUB is among the earliest neuropathological changes in AD (Good et al., 2004). We used the 5xFAD mouse line (Oakley et al., 2006) to study the SUB neural connectivity. The 5xFAD mouse line expresses AD-related human transgenes APP and presenilin 1 (PSEN1), with Swedish (K670N/M671L), Florida (I716V), and London (V717I) mutations in APP and M146L and L286V mutations in PSEN1. 5xFAD mice develop AD-related neuropathological phenotypes early and quickly (Oakley et al., 2006). Anti-amyloid beta (Aβ) immunostaining in a 5xFAD model mouse shows the highest density of Aβ deposits occurring within the pyramidal neurons of the SUB and deep cortical layers (Oakley et al., 2006). To test our hypothesis that SUB input connections are altered in the 5xFAD mouse model, we used genetically modified monosynaptic rabies virus tracing to measure the circuit connectivity of SUB excitatory neurons. Technical developments in rabies tracing methods allow for the semiquantitative mapping of cell-type-specific neural circuit connections (Sun et al., 2014, 2019; Xu et al., 2016, 2020). EnvA pseudotyping enables the modified rabies virus to infect specific cell types. Trans-synaptic rabies spread is limited to direct presynaptic inputs using a glycoprotein gene-deleted (ΔG) rabies virus and transcomplementation with glycoprotein expression offered by helper AAV that provides both tumor virus receptor A (TVA) and glycoprotein in SUB cells.

To determine age-dependent neural circuit alterations, we examined age-matched control wild-type (WT) and 5xFAD mice at two ages (3–4 and 8–9 months) for both sexes. The age groups were chosen based on previous findings of behavioral and social deficits and neuropathological developments (Oakley et al., 2006; Kosel et al., 2019). The 5xFAD mouse SUB shows increased synaptic degeneration and neuron loss at ∼9 months old (Eimer and Vassar, 2013). We are interested in determining the age-related progression and sex-specific differences in the AD phenotype in relation to the SUB input connectivity. Our results show significant alterations in the circuit connectivity of SUB in AD model mice, with overall weaker connectivity strengths, connectivity pattern shifts, and significant sex differences in 5xFAD mice compared with control mice.

## Materials and Methods

### Animals

All experiments were managed according to the National Institutes of Health (NIH) guidelines for animal care and use and were authorized by the University of California, Irvine Institutional Animal Care and Use Committee and Institutional Biosafety Committee. To study SUB circuit connections in the AD mouse, control, and 5xFAD mouse models at two ages were used: WT C57BL/6, with a young group at 3–4 months old (*n* = 8, three males, five females) and a middle-age group at 8–9 months old (*n* = 10, five males, five females), and 5xFAD (C57BL/6 background, The Jackson Laboratory #034848), with a young group at 3–4 months old (*n* = 10, six males, four females) and a middle-age group at 8–9 months old (*n* = 12, five males, seven females). The 5xFAD and WT C57BL/6 mice were of the same genetic background but not littermates. All mice had free access to food and water in their home cages with lights maintained on a 12/12 h light/dark cycle. All personnel working with the rabies virus received rabies vaccinations, and the virus-related experiments were conducted under biosafety level 2 conditions.

### Viral injections

To study neural circuit connections, mice were injected with helper AAVs and rabies virus. The mice were first anesthetized under 1–2% isoﬂurane for 10 min with a 0.8 L/min oxygen flow rate using an isoﬂurane tabletop unit (HME109, Highland Medical Equipment). Their head fur was shaved and the mice were then placed in a rodent stereotaxic frame (Leica Angle Two™ for mouse, Leica Biosystems) with a continuous flow of 1–2% isoflurane anesthetic. After disinfection with 70% alcohol and Betadine, the skin received a small incision, and the skull was exposed to reveal the landmarks of the bregma and lambda. The desired injection site relative to the bregma and lambda was located using a three-axis manipulator, with the guidance of a digital brain atlas of the stereotaxic machine. A small craniotomy was performed above the injection site, exposing the dura. A glass pipette (tip inner diameter, 20–30 μm) was loaded with virus solution and lowered to the target injection site. The virus was delivered through a Picospritzer (Parker Hannifin) pressure injection machine at a rate of 20–30 nl/min with a 10-ms pulse duration. The virus was injected into the dorsal SUB using the following coordinates: anteroposterior (AP), −3.52 mm; mediolateral (ML), +2.50 mm; and dorsoventral (DV), −1.35 mm, all values given relative to the bregma. In order to prevent backflow of the virus immediately following injection, the glass pipette was held at the injection site for 10 min after virus delivery and was then withdrawn at a constant slow speed. After the mice were removed from the stereotaxic frame, their skin was sutured using a tissue adhesive (3 M Vetbond). The mice were injected with 5 mg/kg carprofen subcutaneously to mitigate pain and inflammation and were placed on a heating pad for 15 min postsurgery, where they were monitored until waking, and were then returned to their home cages.

To map the retrograde circuit connectivity of the SUB, the following helper AAVs were used: AAV8-DIO-TC66T-2A-GFP-2A-oG (Salk Institute, 2.36 × 10^13^ GC/ml) and pENN.AAV.CamKII 0.4.Cre.SV40 (Addgene viral prep #105558-AAV1, 5.3 × 10^13^ GC/ml). The pENN.AAV.CamKII 0.4.Cre.SV40 was a gift from Dr. James M. Wilson. The AAV8-DIO-TC66T-2A-GFP-2A-oG was 1:2 diluted with Hanks balanced salt solution (HBSS). The pENN.AAV.CamKII 0.4.Cre.SV40 was 1:4 diluted with HBSS. These two diluted helper AAVs were finally 1:1 mixed. The final titers for the two viruses are 5.9 × 10^12^ GC/ml (AAV8-DIO-TC66T-2A-GFP-2A-oG) and 6.63 × 10^12^ GC/ml (pENN.AAV.CamKII 0.4.Cre.SV40). On the first day, the SUB target site was delivered with 0.05 µl of the AAV mixture by pressure injection. Following 20 d, the mice were injected with the rabies virus EnvA-RV-SADΔG-DsRed [Center for Neural Circuit Mapping (CNCM), 2.1 × 10^9^ IU/ml, 0.4 μl] at the same injection site using pressure injection. The rabies virus was produced at the CNCM virus core facility of the University of California, Irvine, with required cell lines and seeding viruses originally developed by Dr. Edward Callaway's group at the Salk Institute for Biological Studies. For 9 d before the mice were perfused for tissue processing, the rabies virus was allowed to replicate and retrogradely spread from targeted Cre + starter neurons to directly connected presynaptic neurons.

### Histology and immunochemistry

The mice were transcardially perfused with 20 ml of PBS and then with 40 ml of PBS containing 4% paraformaldehyde (PFA) using a mini pump with variable flows (United States Plastic). The perfused brains settled in 4% PFA solution for 24 h followed by 30% sucrose–PBS solution for another 24 h at 4°C before histological processing. The brains were then frozen in dry ice and coronally sectioned at 30 µm thickness on a microtome (Leica SM2010R).

For each mouse brain, coronal sections were collected continuously into a 24-well plate across the wells. For brain-wide neural circuit mapping analysis, one out of three consecutive coronal sections across the entire brain was counterstained with DAPI, mounted, and then imaged. Therefore, a third of the whole brain section series was used for quantification.

To examine Aβ expression, one coronal slice in each mouse for both genders (*n* = 4–5 for each gender) from each mouse group were stained with primary antibody 6E10 (BioLegend, Mouse, #803002, 1:500 dilution), followed by Cy5-conjugated donkey anti-mouse secondary antibody (Jackson ImmunoResearch, #715-175-151, 1:200 dilution).

All sections were imaged by an automated fluorescent slide scanner microscope (Olympus VS120-S6) with a 10× magnification objective. For the high-resolution imaging of selected brain slices, a confocal microscope was utilized (Olympus FLUOVIEW FV3000) with a 40× magnification objective.

### Data quantification and statistical analysis

For amyloid analysis, the amyloid plaque number, plaque size, plaque intensity, intraneuronal 6E10 + number, and SUB field of view were quantified in an unbiased fashion using the measurement tool in Fiji ImageJ (NIH). The SUB region included for amyloid quantification was contralateral to the injection site with similar AP position.

For rabies circuit tracing analysis, we followed an established counting protocol (Sun et al., 2014). A third of the whole brain coronal sections were arranged on an overview canvas in Adobe Photoshop software (Adobe). We identified the brain sections containing the SUB region in which we searched for double-labeled starter neurons with EGFP (helper AAVs) and DsRed (rabies). Next, we aligned the rest of the virally infected brain sections using a standard mouse brain atlas (Paxinos and Franklin, 2008) to determine the anatomical structures of the brain regions containing rabies-labeled neurons using DAPI-labeled morphological features. No stereological measurement protocol was used. All labeled cells in each mounted brain were counted using the Adobe Photoshop count tool. We operationally determined the input connectivity strength index (CSI) as the ratio of the number of presynaptic neurons in a brain region of interest to the number of starter neurons in the SUB region. In this study, the CSI refers to the index that is calculated based on the number of single-labeled red fluorescent neurons in an area versus the number of double-labeled red and green starter neurons in the SUB. The CSI values allow us to numerically compare the input strengths of different brain regions while being normalized to the number of excitatory SUB neurons. We calculated the proportion of input (PI) index as the ratio of the number of labeled presynaptic neurons in a brain region of interest to the total number of labeled neurons in each brain to show the proportional distribution of each input region. For rabies data quantification, only the cases with starter neurons restricted within the dorsal SUB region were included. Any cases that showed evidence of leakage to other adjacent regions were excluded.

The AP CSI distribution curve was plotted to reveal the input pattern across the AP axis of the mouse brains. The input-labeled neuron numbers were counted for every brain slice for each mouse, which corresponds to an AP position number (relative to the bregma) according to the standard mouse brain atlas (Paxinos and Franklin, 2008). The CSI values were calculated as described above. For all mice within the same age/genotype group, the CSI values were registered to similar AP positions and were plotted along the AP axis using a custom-written MATLAB analysis pipeline.

All data are presented as the mean ± SEM. We applied appropriate statistical tests, and the data analysis was conducted using GraphPad Prism (GraphPad Software) or custom-written MATLAB scripts. Statistical analysis methods included the Wilcoxon rank-sum test, paired Wilcoxon rank-sum test, and linear mixed-effects (LME) model (Yu et al., 2022). The LME model was applied to analyze the size and intensity of amyloid plaques. Using the LME model is critical for avoiding false-positive statistical results for comparisons between animal groups where repeated amyloid measurements were taken from each animal. For CSI/PI multiple comparisons across the young and middle-age WT and 5xFAD mice, the *p* values were adjusted for multiple comparisons using the false discovery rate (FDR) Benjamini–Hochberg approach with a desired FDR equals to 0.05. Alpha levels of *p* ≤ 0.05 were considered significant. Different levels of statistical significance are represented by *p ≤ 0.05, **p ≤ 0.01, ***p ≤ 0.001, and ****p ≤ 0.0001.

### Code accessibility

The custom analysis scripts are available on GitHub (https://github.com/QiaoYeNeuro/Brain-Circuit-Connectivity-Analysis).

## Results

### Age-dependent and sex-specific differences in SUB amyloid deposition

**Figure 1.**
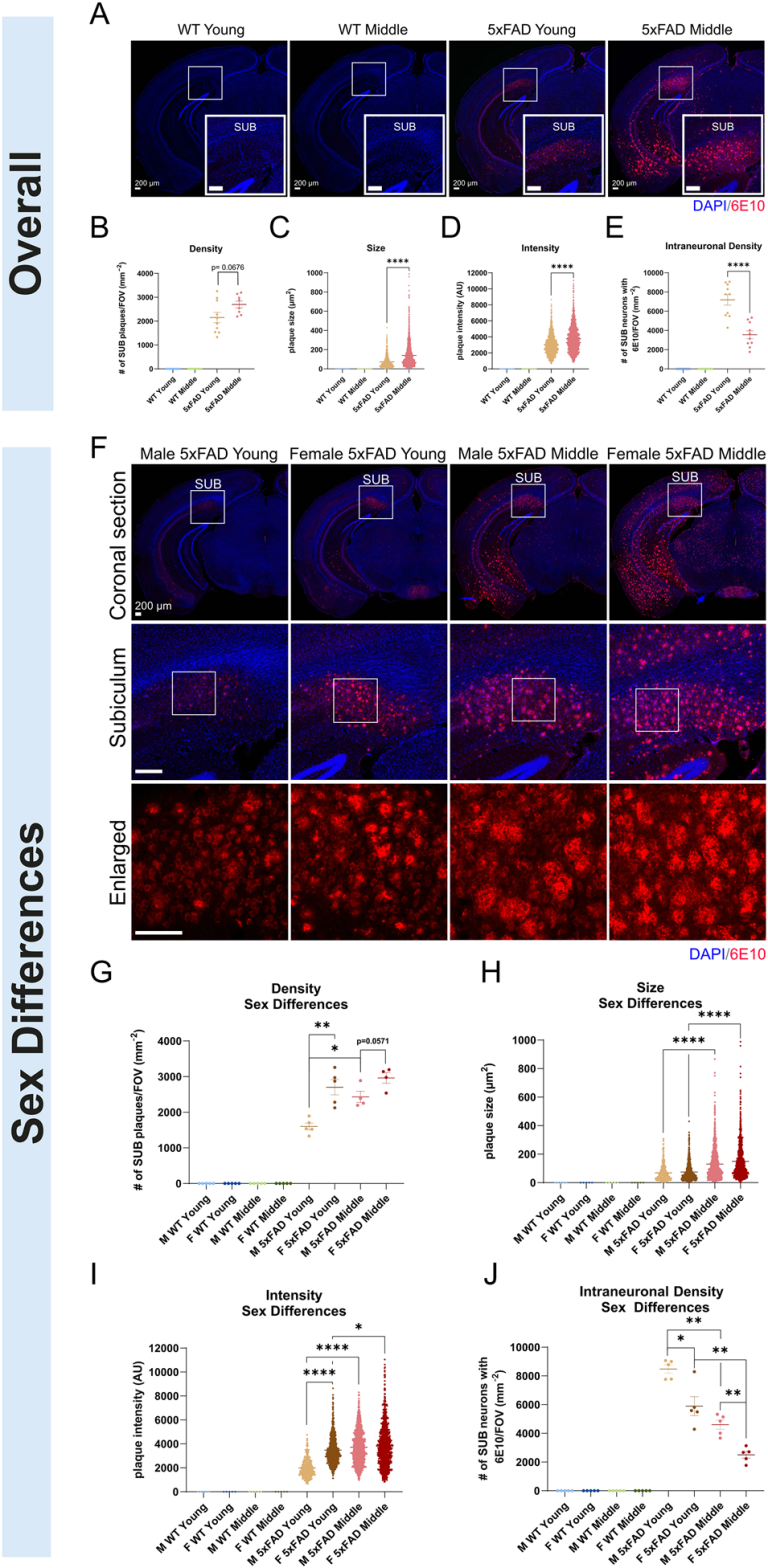
Age-progressive Aβ pathology in 5xFAD mice. 6E10 immunostaining and quantification in age-matched WT and 5xFAD for both ages. ***A***, Representative images of 6E10 amyloid antibody-immunostained plaques and neurons in young and middle age-matched WT and 5xFAD mice. The four panels show coronal brain sections. The large white squares show an enlarged view of the SUB outlined by the smaller white squares. DAPI staining is in blue, and the 6E10 + amyloid signal is in red. ***B–E***, Quantification of extracellular and intraneuronal 6E10 signals in the SUB. More amyloid plaques were detected in 5xFAD middle-age mice relative to 5xFAD young, while none were detected in both age groups of WT mice. 5xFAD middle-age mice show slightly higher plaque density and significantly greater plaque size and intensity relative to 5xFAD young mice (***B***, density, 5xFAD young, 2,151 ± 214.3; 5xFAD middle-age, 2,698 ± 141.8; Wilcoxon rank-sum test, *p* = 0.0676; ***C***, size, 5xFAD young, 73.59 ± 1.645; 5xFAD middle-age, 139.9 ± 2.247; LME model, *p* = 1.86 × 10^−12^; ***D***, intensity, 5xFAD young, 3,018 ± 33.42; 5xFAD middle-age, 3,761 ± 30.54; LME model, *p* = 9.13 × 10^−7^). ***E***, 5xFAD young mice have significantly greater intraneuronal 6E10 density relative to 5xFAD middle-age (density, 5xFAD young, 7,188 ± 548.1; 5xFAD middle-age, 3,554 ± 396.7; Wilcoxon rank-sum test, *p* = 7.58 × 10^−5^). The mouse numbers for each of the four groups of WT and 5xFAD mice of both ages are *n* = 10. ***F–J***, Sex-specific comparison of 6E10 immunostaining. ***F***, Representative images of DAPI and 6E10 antibody immunostaining in 5xFAD mice of both age groups and genders. The first row of panels shows coronal brain sections with DAPI and 6E10 amyloid antibody immunostaining. The second row of panels shows an enlarged view of the SUB outlined by the smaller white squares in the first row. The third row of panels shows an even larger view of the white squares in the second row. ***G–J***, Quantification of sex differences in SUB 6E10-stained amyloid plaques and neurons. ***G***, There is a significant increase in extracellular plaque density in young female 5xFAD and middle-age male 5xFAD compared with male 5xFAD young mice. There is a significant age-dependent increase in extracellular plaque density in male mice, but not in female mice (density, male 5xFAD young, 1,601 ± 94.84; female 5xFAD young, 2,702 ± 214.6; male 5xFAD middle-age, 2,432 ± 157.5; female 5xFAD middle-age, 2,963 ± 148.6; Wilcoxon rank-sum test, male 5xFAD young vs female 5xFAD young, *p* = 0.0079; male 5xFAD young vs male 5xFAD middle-age, *p* = 0.0159; male 5xFAD middle-age vs female 5xFAD middle-age, *p* = 0.0571). ***H***, For both genders of 5xFAD mice, the middle-age mice have significantly greater extracellular plaque size relative to the young mice, revealing that plaque size has an age-dependent increase within the same sex (size, male 5xFAD young, 68.69 ± 2.734; female 5xFAD young, 74.3 ± 2.022; male 5xFAD middle-age, 130.4 ± 2.917; female 5xFAD middle-age, 149.8 ± 3.421; LME model, male 5xFAD young vs male 5xFAD middle-age, *p* = 2.63 × 10^−11^; female 5xFAD young vs female 5xFAD middle-age, *p* = 5.81 × 10^−6^). ***I***, There is a significant age-dependent increase in extracellular plaque intensity within the same sex and a significant sex-dependent increase only in 5xFAD young mice (male 5xFAD young, 1,985 ± 35.98; female 5xFAD young, 3,473 ± 37.11; male 5xFAD middle-age, 3,718 ± 37.67; female 5xFAD middle-age, 3,805 ± 48.52; LME model, male 5xFAD young vs male 5xFAD middle-age, *p* = 1.39 × 10^−23^; female 5xFAD young vs female 5xFAD middle-age, *p* = 0.01184; male 5xFAD young vs female 5xFAD young, *p* = 6.76 × 10^−16^). ***J***, There is a significant sex- and age-dependent decrease in intraneuronal 6E10-stained neuron density in 5xFAD mice. In both male and female 5xFAD mice, middle-age mice have significantly less intraneuronal neuron density than young mice. In 5xFAD mice of the same age group (young or middle), the female mice have significantly less intraneuronal neuron density (density, male 5xFAD young, 8,479 ± 293.9; female 5xFAD young, 5,896 ± 656.4; male 5xFAD middle-age, 4,603 ± 323.5; female 5xFAD middle-age, 2,506 ± 232.0; Wilcoxon rank-sum test, male 5xFAD young vs female 5xFAD young, *p* = 0.0317; male 5xFAD middle-age vs female 5xFAD middle-age, *p* = 0.0079; male 5xFAD young vs male 5xFAD middle-age, *p* = 0.0079; female 5xFAD young vs female 5xFAD middle-age, *p* = 0.0079). The mouse numbers for each of the eight groups (2 age groups, male and female, WT and 5xFAD) are 4–5. One representative section was used for quantification for each mouse brain (Fig. 1*B–E,G–J*). Sample sizes for the LME model are based on both the mouse numbers and the number of individual plaques measured from each mouse brain (Fig. 1*C,D,H,I*; 5xFAD young male, *n* = 432; 5xFAD young female, *n* = 980; 5xFAD middle male, *n* = 1,321; 5xFAD middle female, *n* = 1,255). Scale bars are included in the figure panels. The units for plaque size and plaque density are μm^2^ and per mm^2^, respectively, and the intensity is AU of optical density. Statistical significances are denoted by *p ≤ 0.05, **p ≤ 0.01, ***p ≤ 0.001, and ****p ≤ 0.0001.

In this study, we used the 5xFAD mouse model to study the neural circuit connectivity of SUB excitatory neurons. We focus on the SUB as it is one of the earliest brain regions to show degeneration in AD (Carlesimo et al., 2015). The 5xFAD mouse model expresses five human familial AD-associated genes (Oakley et al., 2006). We first set out to characterize the Aβ level of the SUB region in the 5xFAD mouse model. To detect Aβ levels in 5xFAD and WT mice, we immunostained mouse brain sections with 6E10 Aβ monoclonal antibody (Kim et al., 1988) and quantified the plaque levels in male and female mice at young (3–4 months) and middle (8–9 months) ages for both genotypes (Fig. 1). Aβ antibody 6E10 immunostaining reveals extensive amyloid plaque deposition in the SUB of 5xFAD mice (Fig. 1*A–E*). In contrast, no 6E10-immunopositive staining appears in WT young or middle-age mice. We find staining in two forms, Aβ as plaques in the extracellular space and intraneuronal aggregates. The quantitative plaque analysis of 6E10 staining shows that 5xFAD middle-age mice have significantly greater plaque size and immunofluorescence intensity in the SUB relative to 5xFAD young mice (Fig. 1*C,D*; LME model, *p* = 1.86 × 10^−12^ and *p* = 9.13 × 10^−7^, respectively). The extracellular plaque size value of the 5xFAD young group is 73.59 ± 1.645 and the 5xFAD middle-age group is 139.9 ± 2.247; the extracellular plaque staining intensity value of the 5xFAD young group is 3,018 ± 33.42 and the 5xFAD middle-age group is 3,761 ± 30.54. There are no significant differences detected in the extracellular plaque density between 5xFAD young and middle-age mice, although 5xFAD middle-age mice do have slightly higher plaque density relative to 5xFAD young mice (Fig. 1*B*; Wilcoxon rank-sum test, *p* = 0.0676). The density value of the 5xFAD young-age group is 2,151 ± 214.3 and the 5xFAD middle-age group is 2,698 ± 141.8. While no intraneuronal Aβ staining is present in WT mice, 6E10 immunostaining revealed a substantial density of neurons with intraneuronal staining in 5xFAD mice. The density of neurons with intraneuronal staining in 5xFAD mice has an age-progressive decline, with significantly higher density in 5xFAD young mice compared with 5xFAD middle-age mice, which is consistent with the age-dependent neurodegenerative nature of AD (Fig. 1*E*; Wilcoxon rank-sum test, *p* = 7.58 × 10^−5^). The intraneuronal density value of the 5xFAD young-age group is 7,188 ± 548.1 and the 5xFAD middle-age group is 3,554 ± 396.7. 6E10 immunostaining of the SUB also reveals significant sex differences in plaque density, size, and intensity for 5xFAD mice (Fig. 1*F–J*). Middle-age male and young-age female 5xFAD mice exhibit significantly higher extracellular plaque densities compared with young-age male 5xFAD mice (Fig. 1*G*; Wilcoxon rank-sum test, *p* = 0.0159 and *p* = 0.0079, respectively). The density values of the 5xFAD young group are 1,601 ± 94.84 (male) and 2,702 ± 214.6 (female) and the 5xFAD middle-age group are 2,432 ± 157.5 (male) and 2,963 ± 148.6 (female). In terms of plaque size, significantly larger plaque size is detected in middle-age male 5xFAD mice compared with young male 5xFAD mice, and middle-age female 5xFAD mice show significantly larger plaque size than young female 5xFAD mice (Fig. 1*H*; LME model, *p* = 2.63 × 10^−11^ and *p* = 5.81 × 10^−6^, respectively). The size values of the 5xFAD young group are 68.69 ± 2.734 (male) and 74.30 ± 2.022 (female) and the 5xFAD middle-age group are 130.4 ± 2.917 (male) and 149.8 ± 3.421 (female). Significantly stronger plaque intensity is detected in middle-age male 5xFAD mice compared with young-age male 5xFAD mice, in middle-age female 5xFAD mice compared with young female 5xFAD mice, and in young female 5xFAD young compared with young male 5xFAD mice (Fig. 1*I*; LME model, *p* = 1.39 × 10^−23^, *p* = 0.0118, *p* = 6.76 × 10^−16^, respectively). The intensity values of the 5xFAD young group are 1,985 ± 35.98 (male) and 3,473 ± 37.11 (female) and the 5xFAD middle-age group are 3,718 ± 37.67 (male) and 3,805 ± 48.52 (female). Male and female 5xFAD mice exhibit a significant decrease in the density of neurons with intraneuronal staining as age progresses, and the neuronal density of young and middle-age male 5xFAD mice is significantly higher than that of young and middle-age 5xFAD female mice, respectively (Fig. 1*J*; LME model, *p* = 0.0079, *p* = 0.0079, *p* = 0.0317, and *p* = 0.0079, respectively). The intraneuronal density values of the 5xFAD young group are 8,579 ± 293.9 (male) and 5,896 ± 656.4 (female) and the 5xFAD middle-age group are 4,603 ± 323.5 (male) and 2,506 ± 232 (female). For each of the four groups, the sample size is *n* = 4–5 mice. One representative section was quantified from each mouse (Fig. 1*B–E,G–J*). Sample sizes for the LME model analysis consist of the number of individual plaques for each group (Fig. 1*C,D,H,I*; extracellular: 5xFAD young male, *n* = 432; 5xFAD young female, *n* = 980; 5xFAD middle male, *n* = 1,321; 5xFAD middle female, *n* = 1,255). More statistical details are provided in Tables 1 and 2. The units for plaque size and plaque density are μm^2^ and per mm^2^, respectively, and the intensity is arbitrary unit (AU) of optical density. Overall, our SUB amyloid immunostaining results reveal strong age-dependent and sex-dependent alterations.

**Table 1.** Summary of quantification for 6E10 Aβ immunostaining

Summary	WT young		WT middle		5xFAD young		5xFAD middle	
	# of mice: 8–10 for each group							
	Mean	SEM	Mean	SEM	Mean	SEM	Mean	SEM
Density (mm^−2^)	0	0	0	0	2,151	±214.3	2,698	±141.8
Size (μm^2^)	0	0	0	0	73.59	±1.645	139.9	±2.247
Intensity (AU)	0	0	0	0	3,018	±33.42	3,761	±30.54
Intracellular density (mm^−2^)	0	0	0	0	7,188	±548.1	3,554	±396.7
Statistical comparison								
Comparison	5xFAD young versus 5xFAD middle							
	*p* value				Significance			
Density (mm^−2^)	6.7600 × 10^−2^				ns			
Size (μm^2^)	1.8648 × 10^−12^				****			
Intensity (AU)	9.1253 × 10^−7^				****			
Intracellular density (mm^−2^)	7.5776 × 10^−5^				****			

The 6E10 amyloid staining quantification results and statistical comparisons summary for WT young, WT middle-age, 5xFAD young, and 5xFAD middle-age mice (*n* = 8–10 for each group). Statistical significances are denoted by *p ≤ 0.05, **p ≤ 0.01, ***p ≤ 0.001, and ****p ≤ 0.0001. Statistical method: Wilcoxon rank-sum test and LME model.

**Table 2.** Summary of quantification for sex-specific 6E10 Aβ immunostaining

Summary	WT young male		WT young female		WT middle male		WT middle female		5xFAD young male		5xFAD young female		5xFAD middle male		5xFAD middle female	
	# of mice: 4–5 for each group															
	Mean	SEM	Mean	SEM	Mean	SEM	Mean	SEM	Mean	SEM	Mean	SEM	Mean	SEM	Mean	SEM
Density (mm^−2^)	0	0	0	0	0	0	0	0	1,601	94.8	2,702	214.6	2,432	157.5	2,963	148.6
Size (μm^2^)	0	0	0	0	0	0	0	0	68.7	2.7	74.3	2.0	130	2.9	150	3.4
Intensity (AU)	0	0	0	0	0	0	0	0	1,985	36.0	3,473	37.1	3,718	37.7	3,805	48.5
Intracellular density (mm^−2^)	0	0	0	0	0	0	0	0	8,479	293.9	5,896	656.4	4,603	323.5	2,506	232.0
Statistical comparison																
Comparison	5xFAD young male versus 5xFAD young female				5xFAD middle male versus 5xFAD middle female				5xFAD young male versus 5xFAD middle male				5xFAD young female versus 5xFAD middle female			
	*p* value		Significance		*p* value		Significance		*p* value		Significance		*p* value		Significance	
Density (mm^−2^)	7.9000 × 10^−3^		**		5.7100 × 10^−2^		ns		1.5900 × 10^−2^		*		4.1270 × 10^−1^		ns	
Size (μm^2^)	7.7752 × 10^−1^		ns		2.0914 × 10^−1^		ns		2.6272 × 10^−11^		****		5.8125 × 10^−6^		****	
Intensity (AU)	6.7646 × 10^−16^		****		3.4272 × 10^−1^		ns		1.3916 × 10^−23^		****		1.1840 × 10^−2^		*	
Intracellular density (mm^−2^)	3.1700 × 10^−2^		*		7.9000 × 10^−3^		**		7.9000 × 10^−3^		**		7.9000 × 10^−3^		**	

The 6E10 amyloid staining quantification results and statistical comparisons summary for both sexes of WT young, WT middle-age, 5xFAD young, and 5xFAD middle-age mice (*n* = 4–5 for each subgroup). Statistical significances are denoted by *p ≤ 0.05, **p ≤ 0.01, ***p ≤ 0.001, and ****p ≤ 0.0001. Statistical method: Wilcoxon rank-sum test and LME model.

### Brain-wide neural circuit connections to SUB excitatory cells are altered in 5xFAD mice

**Figure 2.**
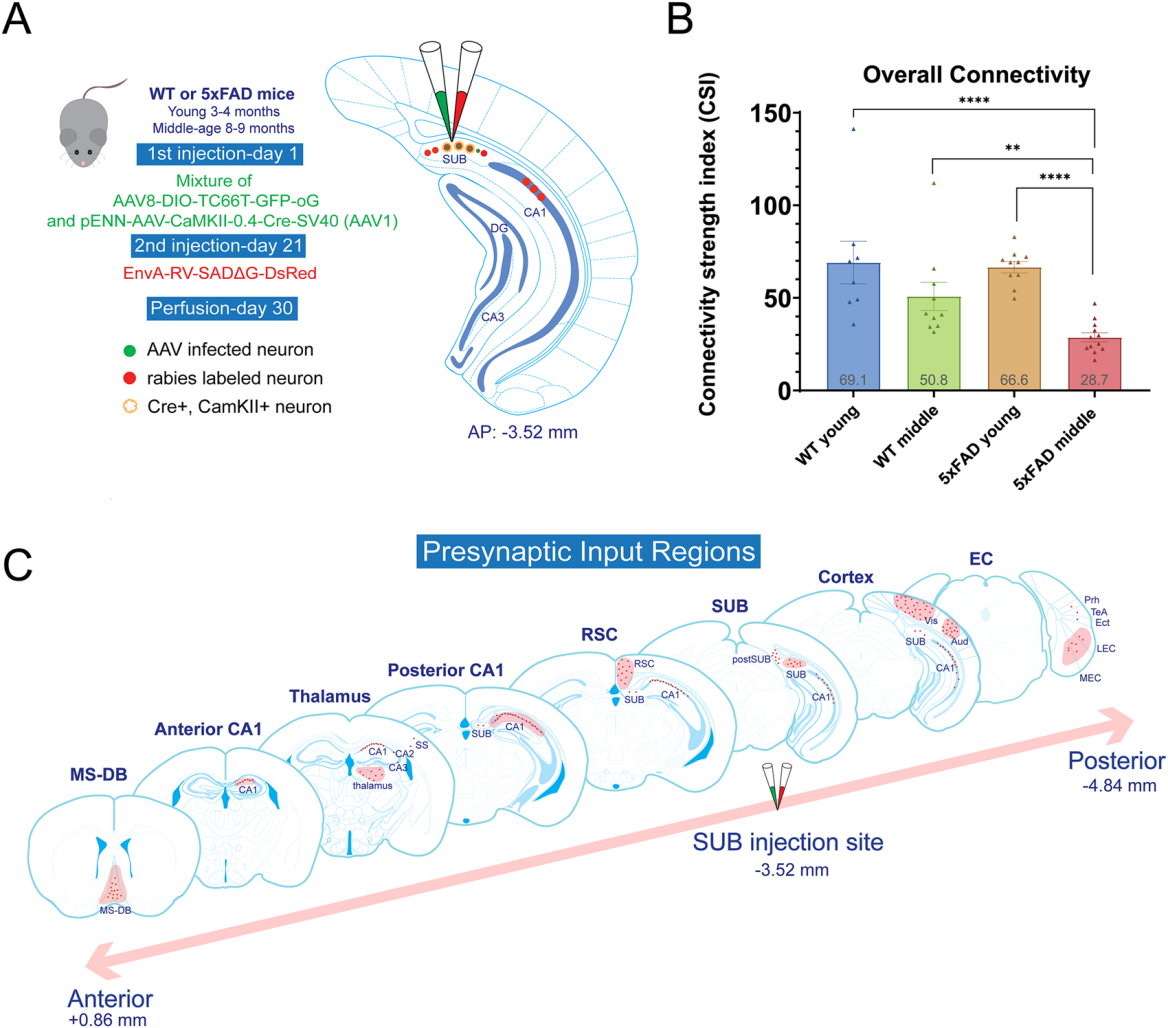
Brain-wide presynaptic inputs to SUB excitatory neurons revealed by monosynaptic rabies tracing. ***A***, Schematic of cell-type-specific retrograde monosynaptic rabies tracing. To specifically label excitatory SUB neurons, the helper AAV virus (AAV-DIO-TC66T-GFP-oG and AAV-CaMKII-Cre), labeled green, was injected into the dorsal SUB, followed 20 d later at the same site by an injection of rabies virus (EnvA-RV-SADΔG-DsRed), labeled red. The neurons labeled both green and red represent the starter neurons. The neurons labeled only red represent the presynaptic inputs to the starter neurons. Nine days after rabies injection, the mice were perfused for further analysis. ***B***, Overall brain CSI for WT young (*n* = 8), WT middle-age (*n* = 10), 5xFAD young (*n* = 10), and 5xFAD middle-age (*n* = 12) groups (69.1 ± 11.5, 50.8 ± 7.6, 66.6 ± 3.1, and 28.7 ± 2.5, respectively). The CSI is defined as the number of input neurons normalized by the number of starter neurons. The 5xFAD middle-age group has a significantly lower CSI than the WT young, WT middle-age, and 5xFAD young groups (Wilcoxon rank-sum test, *p* = 6.35 × 10^−5^, *p* = 1.13 × 10^−3^, and *p* = 3.09 × 10^−6^, respectively). Data are presented as mean ± SEM. Statistical significances are denoted by *p ≤ 0.05, **p ≤ 0.01, ***p ≤ 0.001, and ****p ≤ 0.0001. ***C***, Coronal sections of major input regions to SUB excitatory neurons arranged along the AP axis, including MS-DB, anterior and posterior CA1, thalamus, RSC, SUB, Vis cortex, Aud cortex, and EC. The major input regions are labeled in red-shaded areas. Other input regions such as CA2, CA3, postSUB, SS cortex, Prh, TeA, and Ect are labeled as well. The injection site is denoted along the AP axis. Red color labels represent presynaptic input neurons.

To investigate whether the SUB circuit connectivity is altered in AD model mice, we applied Cre-dependent monosynaptic rabies tracing in gender-balanced, age-matched 5xFAD and WT mice as shown schematically (Fig. 2*A*). The rabies tracing was performed at young (3–4 months) and middle (8–9 months) ages. The Cre-dependent monosynaptic rabies virus enables retrograde mapping of neural circuit inputs to specific neuron cell types in conjunction with helper AAVs. Our monosynaptic rabies tracing approach takes advantage of the ability to target rabies infection to specific cell types using EnvA pseudotyping, and to limit trans-synaptic spread to direct inputs, by using glycoprotein gene-deleted (ΔG) rabies virus and transcomplementation. EnvA-pseudotyped rabies virus can only infect neurons that express avian TVA, an avian receptor protein that is absent in mammalian cells unless it is provided through exogenous gene delivery. The deletion-mutant rabies virus can then be transcomplemented with the expression of rabies glycoprotein in the same TVA-expressing cells to enable its retrograde spread restricted to direct presynaptic neurons. To target the excitatory neurons in the SUB, a mixture of helper AAV was injected (AAV1-CaMKIIα-cre and AAV8-DIO-TC66T-GFP-oG) stereotaxically into the SUB in the right hemisphere of all experimental mice. The combination of the double inverted open (DIO) frame and Cre recombinase allows for the expression of a highly specific TVA receptor (TC66T; Miyamichi et al., 2013) and optimized rabies glycoprotein (oG) in CaMKIIα-positive excitatory SUB neurons. Twenty days after the initial AAV injection, the EnvA-pseudotyped G-deleted rabies virus (EnvA-RV-SADΔG-DsRed) was delivered stereotaxically to the same injection site as the helper AAV. The rabies virus was incubated for 9 d before the mouse brains were harvested for sectioning and further histological processing. Overall, we map the presynaptic connections of SUB excitatory neurons in multiple brain regions for both 5xFAD and WT mice (Fig. 2*C*). These inputs primarily originate from the MS-DB; hippocampal CA1, CA2, and CA3; retrosplenial cortex (RSC); SUB; postsubiculum (postSUB); visual (Vis) cortex; auditory (Aud) cortex; somatosensory (SS) cortex; temporal association cortex (TeA); perirhinal cortex (Prh); ectorhinal cortex (Ect); lateral entorhinal cortex (LEC); and medial entorhinal cortex (MEC; Fig. 2*C*). Notably, the SUB excitatory neurons receive strong intrinsic connections from local SUB neurons. For both 5xFAD and WT mice at young and middle ages, the strongest inputs originate from the hippocampal CA1 pyramidal layer (CA1_py)­. To quantitatively assess the brain-wide overall connectivity of SUB excitatory neurons, we divide the total number of neurons labeled in the entire brain over the number of starter neurons. The initial AAV and rabies-infected neurons are defined as the starter neurons, which express both EGFP and DsRed signals; rabies-labeled presynaptic neurons display only DsRed fluorescence (Fig. 2*A*). Our results indicate that the SUB excitatory neuron input connections in middle-age 5xFAD mice are compromised with weaker overall brain-wide connectivity compared with WT mice at both ages and young 5xFAD mice (Fig. 2*B*).

### Regional-specific SUB neural circuit connectivity alterations in 5xFAD mice

**Figure 3.**
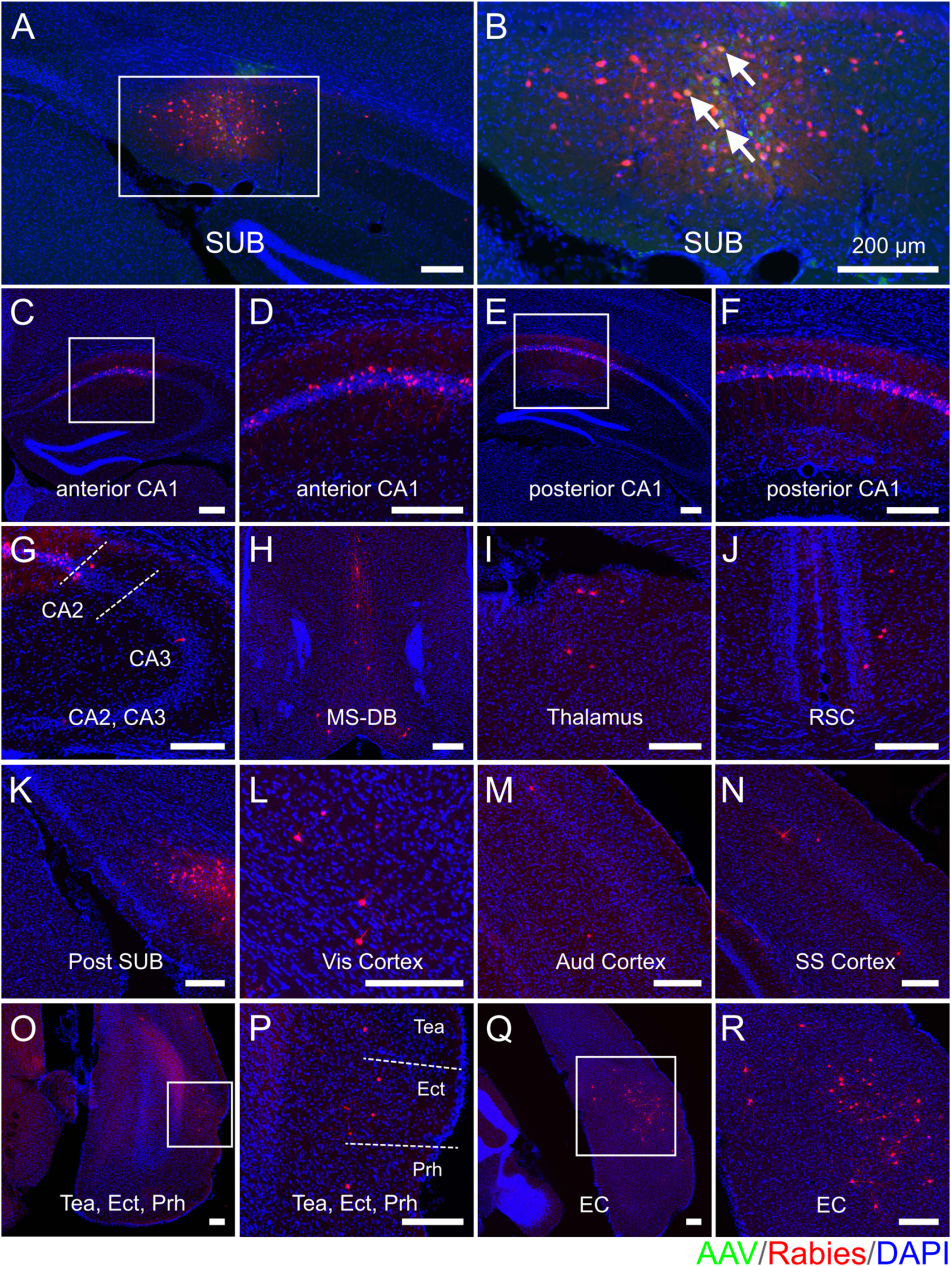
Monosynaptic rabies tracing maps region-specific inputs to excitatory SUB cells in young WT mice. Representative fluorescent coronal section images from the WT young group. ***A***, Dorsal SUB injection site. Rabies virus-infected neurons are labeled by DsRed, and AAV-infected neurons are labeled by EGFP. ***B***, Enlarged view of the SUB injection site, including white arrows to represent starter neurons. Starter neurons are labeled both red (RV) and green (AAV) fluorescent proteins. ***C–R***, Major presynaptic input regions of the SUB mapped by rabies virus-mediated retrograde monosynaptic tracing. The input regions include the SUB (***A,B***), anterior CA1 (***C,D***), posterior CA1 (***E,F***), CA2 and CA3 (***G***), MS-DB (***H***), thalamus (***I***), RSC (***J***), postSUB (***K***), Vis cortex (***L***), Aud cortex (***M***), SS cortex (***N***), Tea and Ect and Prh (***O,P***), and EC (***Q,R***). (***D***), (***F***), (***P***), and (***R***) are enlarged views of the white boxes in (***C***), (***E***), (***O***), and (***Q***), respectively. All slices are counterstained by DAPI in blue. Scale bars labeled for each panel represent 200 μm.

**Figure 4.**
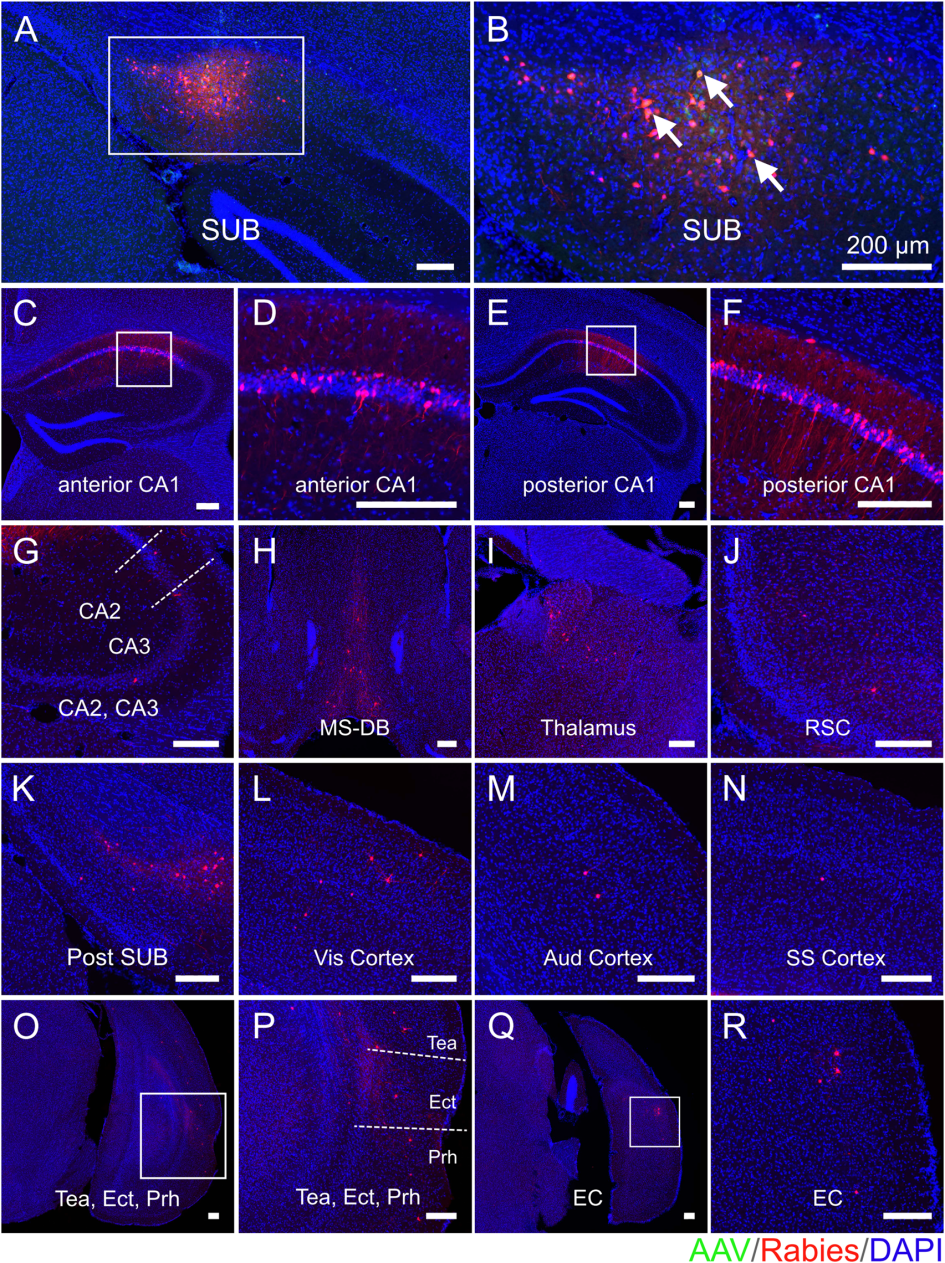
Monosynaptic rabies tracing maps region-specific inputs to excitatory SUB cells in middle-age WT mice. Representative fluorescent coronal section images from the WT middle-age group. ***A***, Dorsal SUB injection site. Rabies virus-infected neurons are labeled by DsRed, and AAV-infected neurons are labeled by EGFP. ***B***, Enlarged view of the SUB injection site, including white arrows to represent starter neurons. Starter neurons are labeled both red (RV) and green (AAV) fluorescent proteins. ***C–R***, Major presynaptic input regions of the SUB mapped by rabies virus-mediated retrograde monosynaptic tracing. The input regions include the SUB (***A,B***), anterior CA1 (***C,D***), posterior CA1 (***E,F***), CA2 and CA3 (***G***), MS-DB (***H***), thalamus (***I***), RSC (***J***), postSUB (***K***), Vis cortex (***L***), Aud cortex (***M***), SS cortex (***N***), Tea and Ect and Prh (***O,P***), and EC (***Q,R***). (***D***), (***F***), (***P***), and (***R***) are enlarged views of the white boxes in (***C***), (***E***), (***O***), and (***Q***), respectively. All slices are counterstained by DAPI in blue. Scale bars labeled for each panel represent 200 μm.

**Figure 5.**
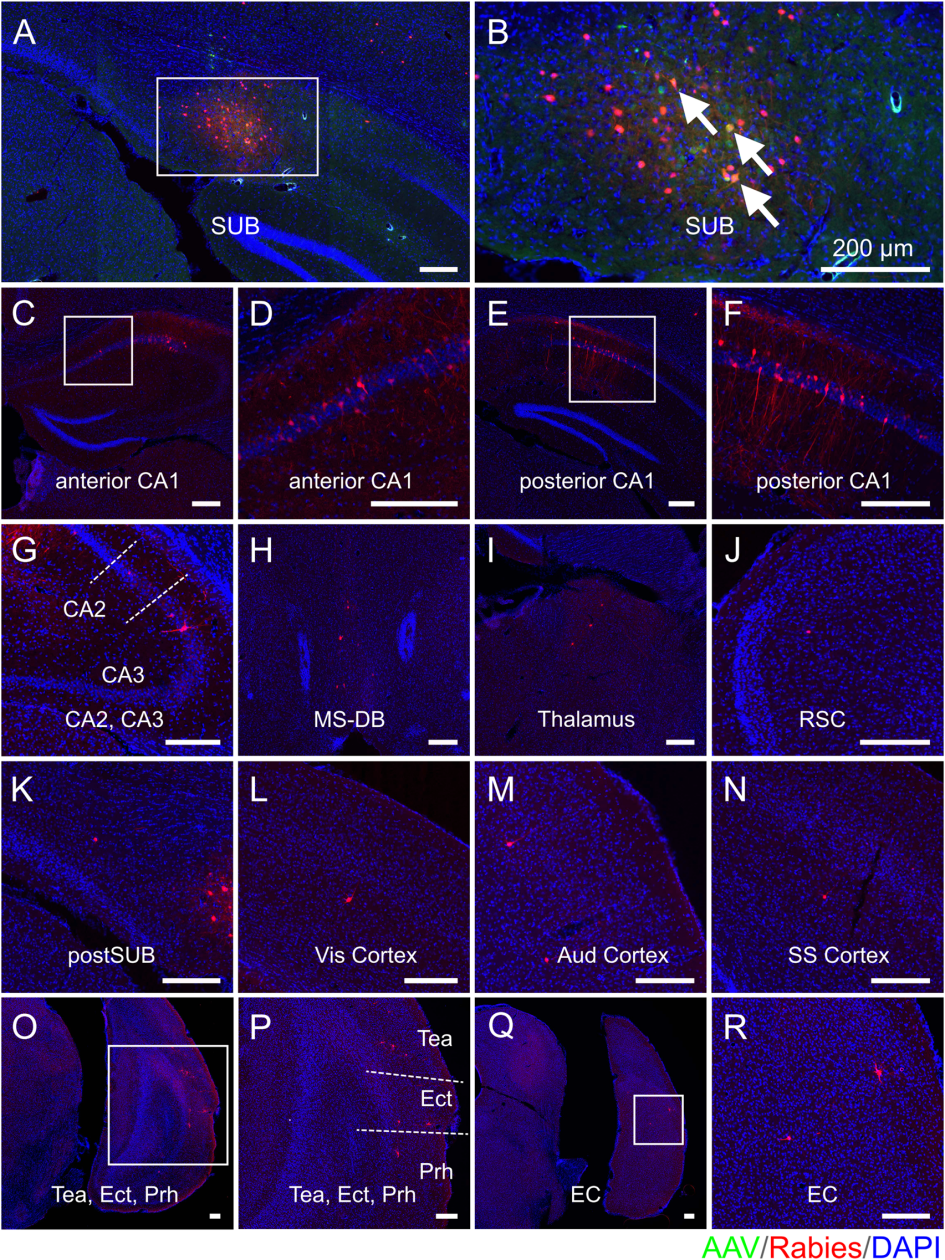
Monosynaptic rabies tracing maps region-specific inputs to excitatory SUB cells in young 5xFAD mice. Representative fluorescent coronal section images from the 5xFAD young group. ***A***, Dorsal SUB injection site. Rabies virus-infected neurons are labeled by DsRed, and AAV-infected neurons are labeled by EGFP. ***B***, Enlarged view of the SUB injection site, including white arrows to represent starter neurons. Starter neurons are labeled both red (RV) and green (AAV) fluorescent proteins. ***C–R***, Major presynaptic input regions of the SUB mapped by rabies virus-mediated retrograde monosynaptic tracing. The input regions include the SUB (***A,B***), anterior CA1 (***C,D***), posterior CA1 (***E,F***), CA2 and CA3 (***G***), MS-DB (***H***), thalamus (***I***), RSC (***J***), postSUB (***K***), Vis cortex (***L***), Aud cortex (***M***), SS cortex (***N***), Tea and Ect and Prh (***O,P***), and EC (***Q,R***). (***D***), (***F***), (***P***), and (***R***) are enlarged views of the white boxes in (***C***), (***E***), (***O***), and (***Q***), respectively. All slices are counterstained by DAPI in blue. Scale bars labeled for each panel represent 200 μm.

**Figure 6.**
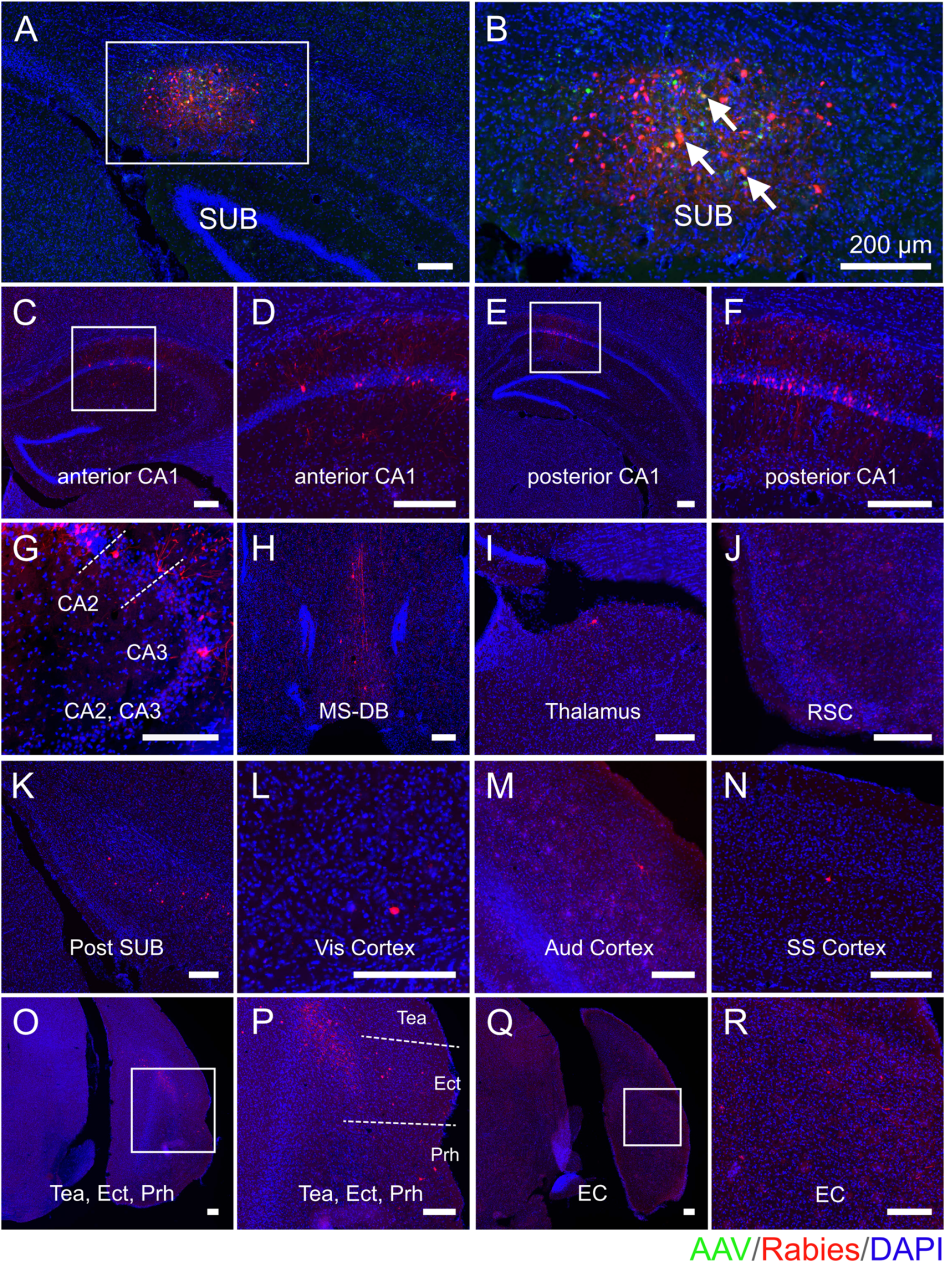
Monosynaptic rabies tracing maps region-specific inputs to excitatory SUB cells in middle-age 5xFAD mice. Representative fluorescent coronal section images from 5xFAD middle-age group. ***A***, Dorsal SUB injection site. Rabies virus-infected neurons are labeled by DsRed, and AAV-infected neurons are labeled by EGFP. ***B***, Enlarged view of the SUB injection site, including white arrows to represent starter neurons. Starter neurons are labeled both red (RV) and green (AAV) fluorescent proteins. ***C–R***, Major presynaptic input regions of the SUB mapped by rabies virus-mediated retrograde monosynaptic tracing. The input regions include the SUB (***A,B***), anterior CA1 (***C,D***), posterior CA1 (***E,F***), CA2 and CA3 (***G***), MS-DB (***H***), thalamus (***I***), RSC (***J***), postSUB (***K***), Vis cortex (***L***), Aud cortex (***M***), SS cortex (***N***), Tea and Ect and Prh (***O,P***), and EC (***Q,R***). (***D***), (***F***), (***P***), and (***R***) are enlarged views of the white boxes in (***C***), (***E***), (***O***), and (***Q***), respectively. All slices are counterstained by DAPI in blue. Scale bars labeled for each panel represent 200 μm.

To map and compare the regional-specific connections to SUB excitatory neurons in age-matched 5xFAD and WT mice, one out of every three sections in each brain coronal section series was mounted for examination and quantification of starter cells and their presynaptic cells in different brain structures. The example presynaptic-labeled brain regions as well as the SUB injection site are shown for each mouse group (WT young, Fig. 3; WT middle-age, Fig. 4; 5xFAD young age, Fig. 5; 5xFAD middle-age, Fig. 6). All brain slices are counterstained with DAPI. Neurons infected with AAV are shown in green (GFP) and rabies-labeled neurons are shown in red (DsRed). For both WT and 5xFAD mice at young and middle ages, the SUB presynaptic input regions include the ipsilateral hippocampal CA1_py, CA1 oriens layer (CA1_or), CA1 radiatum layer (CA1_rad), CA1 lacunosum-moleculare layer (CA1_lmol), CA2 pyramidal layer (CA2_py), CA2 oriens layer (CA2_or), CA3 pyramidal layer (CA3_py), CA3 oriens layer (CA3_or), local SUB neurons, postSUB, MS-DB, thalamus, RSC, Vis cortex, Aud cortex, SS cortex, TeA, Prh, Ect, LEC, and MEC. Very sparse labeling was occasionally observed in the contralateral CA1, hypothalamus, dentate gyrus, cingulate cortex, and midbrain and was thus not included in statistical comparison analysis. The SUB input brain regions mapped by rabies tracing in WT young, WT middle-age, 5xFAD young, and 5xFAD middle-age are generally consistent.

To quantitatively compare the connectivity difference across different experiment groups, we aligned one-third of the entire brain coronal slices and quantified the starter neuron numbers and the presynaptic input neuron numbers for each brain region. To determine semiquantitative normalized neural connectivity, we operationally defined the CSI. The CSI is presented by the number of rabies-labeled presynaptic neurons in a brain region divided by the number of starter neurons in the SUB. The CSI value reflects an absolute measurement of the connectivity between the SUB and its presynaptic input regions.

**Figure 7.**
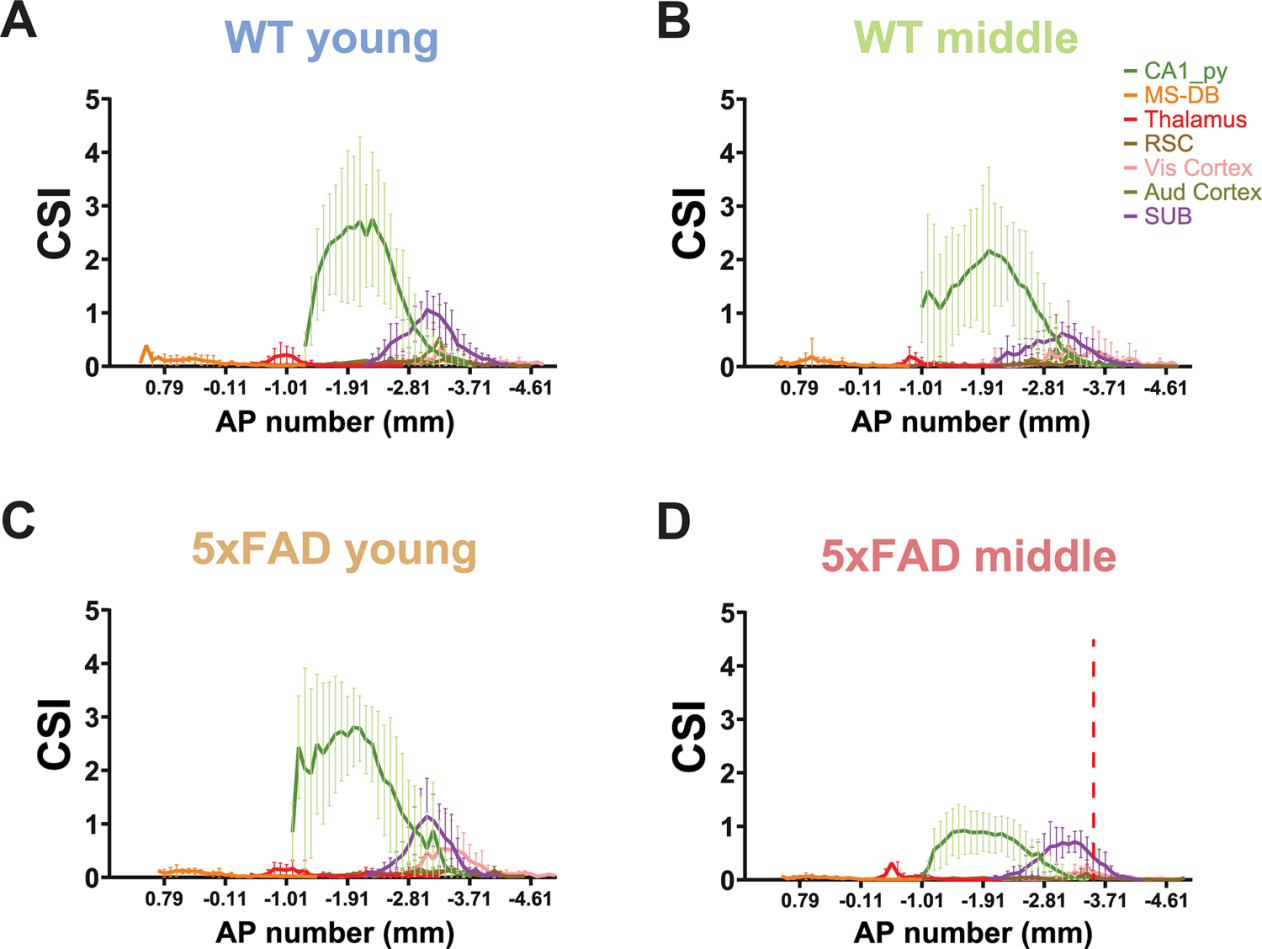
AP CSI distributions across the brain for different age and genotype groups. The CSI is defined as the number of input neurons normalized by the number of starter neurons; the AP positions are given relative to bregma values. Representative input regions were used for the AP plot, including the CA1_py layer, MS-DB, thalamus, RSC, Vis cortex, Aud cortex, and SUB. ***A***, WT young group (*n* = 8). ***B***, WT middle-age group (*n* = 10). ***C***, 5xFAD young group (*n* = 10). ***D***, 5xFAD middle-age group (*n* = 12). The red dashed line at AP = −3.52 mm shows the position of the viral injection site. There is a significant difference in the CA1_py distribution between WT young and 5xFAD middle-age groups (paired Wilcoxon rank-sum test, *p* = 1.67 × 10^−6^).

To investigate the brain-wide neuron input distribution pattern along the AP axis in both WT and 5xFAD mice at young and middle ages, the input CSI values of major brain regions are calculated for each brain section along the AP axis of the mouse brain (Fig. 7). The example brain regions included in the analysis are the ipsilateral hippocampal CA1_py, SUB, MS-DB, RSC, Vis cortex, and Aud cortex. The SUB excitatory cells in the four groups of mice show an overall similar distribution pattern of regional inputs, with MS-DB accounting for the most anterior position, followed by the thalamus, hippocampal CA1, Vis cortex, and Aud cortex, moving progressively more posteriorly. Notably, for 5xFAD middle-age mice, there is a significant decrease in the area under curve of CA1 pyramidal inputs compared with the WT young group (5xFAD middle-age vs WT young, *p* = 1.67 × 10^−6^, paired Wilcoxon rank-sum test).

### Quantitative comparisons of SUB connectivity strengths show significant age-dependent differences between controls versus 5xFAD mice

**Figure 8.**
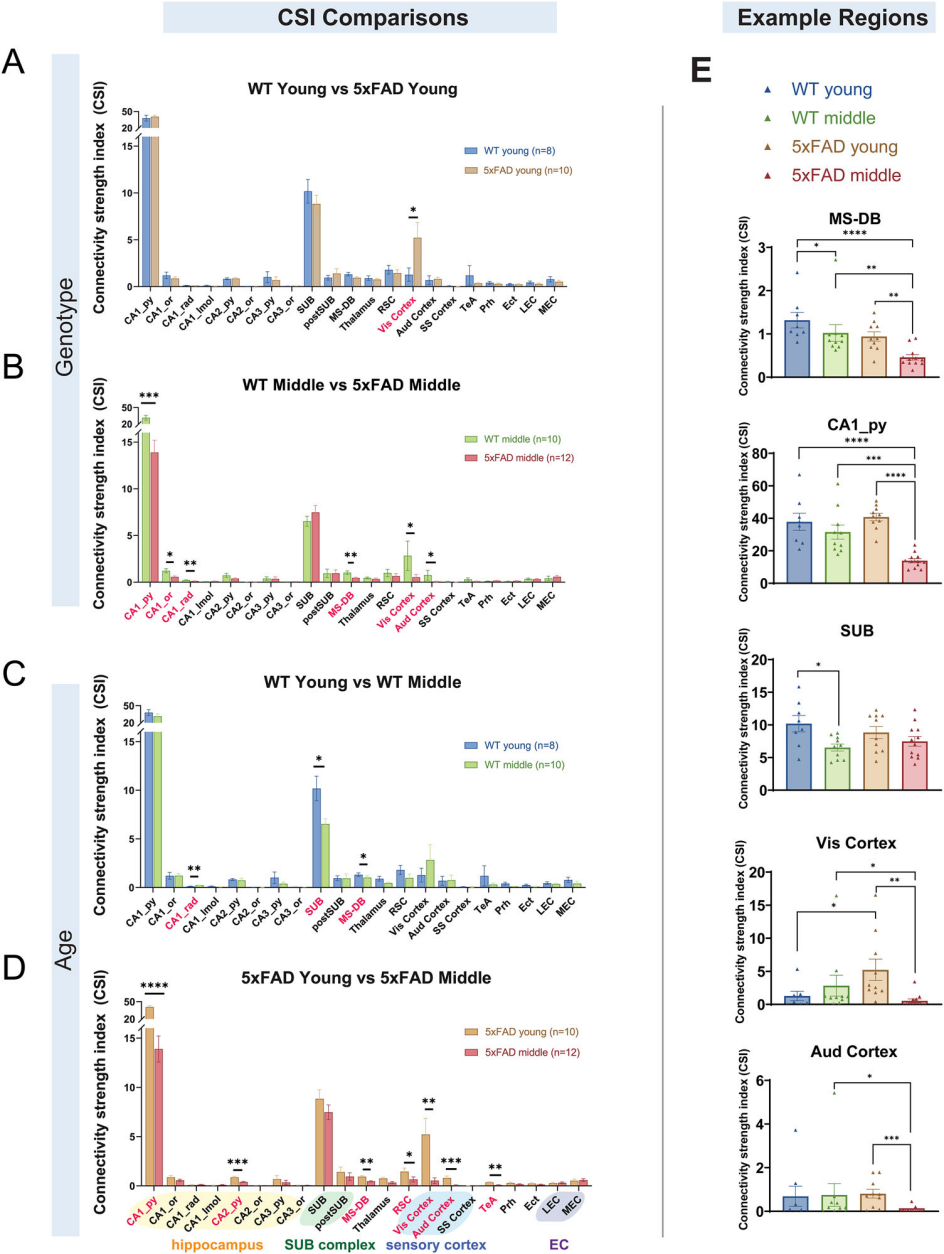
Connectivity strength quantification of presynaptic input regions to SUB excitatory neurons. The CSI is the ratio of the SUB input neuron number in a subregion to the total starter neuron number in a brain. The input regions include the hippocampal CA1, CA2, and CA3 sublayers, as well as the SUB, postSUB, thalamus, RSC, Vis cortex, Aud cortex, SS cortex, TeA, Prh, Ect, LEC, and MEC. The regions showing statistical differences are shown in red text. ***A***, WT young mice have significantly lower CSI values relative to 5xFAD young mice in the Vis cortex (*p* = 1.29 × 10^−2^). ***B***, WT middle-age mice have significantly higher CSI values relative to 5xFAD middle-age mice in the CA1_py, CA1_or, CA1_rad, MS-DB, Vis cortex, and Aud cortex (*p* = 1.2 × 10^−4^, *p* = 4.46 × 10^−2^, *p* = 8.78 × 10^−3^, *p* = 3.33 × 10^−3^, *p* = 1.29 × 10^−2^, and *p* = 1.38 × 10^−2^, respectively). ***C***, WT young mice have significantly higher CSI values relative to WT middle-age mice in the SUB and MS-DB (*p* = 4.66 × 10^−2^, and *p* = 2.74 × 10^−2^, respectively). WT middle-age mice have significantly higher CSI values relative to WT young mice in the CA1_rad (*p* = 8.78 × 10^−3^). ***D***, 5xFAD young mice have significantly higher CSI values relative to 5xFAD middle-age mice in the CA1_py, CA2_py, MS-DB, RSC, Vis cortex, Aud cortex, and TeA (*p* = 1 × 10^−5^, *p* = 5.6 × 10^−4^, *p* = 4.03 × 10^−3^, *p* = 4.46 × 10^−2^, *p* = 1.2 × 10^−3^, *p* = 8.1 × 10^−4^, and *p* = 6.45 × 10^−3^, respectively). ***E***, CSI comparisons of example regions across four groups of mice. Representative regions include the MS-DB, CA1_py, SUB, Vis Cortex, and Aud Cortex. The mouse numbers for each group are WT young, *n* = 8; WT middle-age, *n* = 10; 5xFAD young, *n* = 10; and 5xFAD middle-age, *n* = 12. The statistical test is the Wilcoxon rank-sum test. The *p* values are adjusted with the FDR Benjamini–Hochberg approach (desired FDR = 0.05). All data are represented with mean ± SEM. Statistical significances are denoted by *p ≤ 0.05, **p ≤ 0.01, ***p ≤ 0.001, and ****p ≤ 0.0001. See also Tables 3 and 4.

To quantitatively assess the connectivity strength differences for WT young, WT middle-age, 5xFAD young, and 5xFAD middle-age mice, we calculated the CSI values for each presynaptic brain region and compared them across genotypes and ages (Fig. 8, Table 3). The presynaptic input regions include subregions within the hippocampus, SUB complex, sensory cortex, and EC, as denoted by colored shades, as well as other regions mentioned above (Fig. 8*D*). Overall, the SUB receives the strongest inputs from the ipsilateral CA1_py for all mouse groups. As noted, the average CSI value of CA1_py inputs is very extensive (∼38 for WT young mice). Besides the strongest inputs from CA1 sublayers, the rabies tracing reveals substantial noncanonical connections from CA2 and CA3. The second largest set of inputs originates from local SUB neurons. Among the sensory cortical regions, the Vis cortex is the major input source. For the thalamic inputs, the inputs are primarily from the anterior thalamus.

**Table 3.** Data summary of CSI and PI measurements

	WT young		WT middle		5xFAD young		5xFAD middle	
	Mean	SEM	Mean	SEM	Mean	SEM	Mean	SEM
CSI summary								
CA1_py	37.7953	5.2921	31.5000	4.3837	40.7620	2.3022	13.8835	1.3216
CA1_or	1.2013	0.3422	1.2242	0.2206	0.8664	0.1782	0.5687	0.1026
CA1_rad	0.1160	0.0193	0.2302	0.0286	0.0985	0.0314	0.1216	0.0318
CA1_lmol	0.1093	0.0401	0.0628	0.0147	0.0632	0.0247	0.1024	0.0263
CA2_py	0.8291	0.0965	0.7376	0.2225	0.8725	0.0868	0.4024	0.0506
CA2_or	0.0296	0.0093	0.0234	0.0072	0.0583	0.0127	0.0438	0.0130
CA3_py	1.0087	0.5901	0.3927	0.1854	0.6763	0.3820	0.3453	0.2308
CA3_or	0.0475	0.0128	0.0413	0.0074	0.0748	0.0133	0.0686	0.0112
SUB	10.1783	1.2574	6.5282	0.5425	8.8363	0.9168	7.4721	0.7437
PostSUB	0.9515	0.2478	0.9427	0.4536	1.4021	0.5041	0.9485	0.3778
MS-DB	1.3175	0.1816	1.0204	0.1924	0.9410	0.1071	0.4593	0.0628
Thalamus	0.9022	0.2451	0.4653	0.0931	0.7509	0.1263	0.3354	0.1102
RSC	1.7979	0.4588	0.9879	0.3911	1.4492	0.3497	0.6619	0.2547
Vis cortex	1.2750	0.7136	2.8280	1.5745	5.2217	1.6207	0.5301	0.2877
Aud Cortex	0.6859	0.4599	0.7486	0.5241	0.8048	0.2012	0.0672	0.0390
SS Cortex	0.0676	0.0329	0.0575	0.0416	0.0221	0.0078	0.0224	0.0123
TeA	1.1864	1.0377	0.3050	0.1541	0.3566	0.0686	0.0888	0.0291
Prh	0.4073	0.1505	0.1088	0.0256	0.2884	0.0872	0.1620	0.0475
Ect	0.2519	0.0940	0.0863	0.0268	0.2362	0.0678	0.1280	0.0474
LEC	0.4429	0.1457	0.3485	0.0905	0.2719	0.1078	0.3179	0.0902
MEC	0.7852	0.2759	0.4092	0.2281	0.5234	0.1567	0.5871	0.1493
PI summary								
CA1_py	58.4838	3.1812	64.8701	2.4048	63.0325	1.8142	51.0676	2.1452
CA1_or	1.9183	0.4506	2.6308	0.4665	1.3955	0.2988	2.0883	0.3278
CA1_rad	0.2115	0.0556	0.5114	0.0625	0.1582	0.0507	0.4526	0.0978
CA1_lmol	0.1877	0.0578	0.1572	0.0393	0.1127	0.0514	0.4002	0.1152
CA2_py	1.4048	0.1801	1.4068	0.2230	1.4031	0.1889	1.5151	0.2089
CA2_or	0.0518	0.0136	0.0575	0.0171	0.0980	0.0227	0.1640	0.0518
CA3_py	1.7384	0.9727	1.1501	0.6257	1.2584	0.7657	1.3853	1.0092
CA3_or	0.0716	0.0149	0.0992	0.0186	0.1258	0.0258	0.2486	0.0324
SUB	16.8789	1.8477	14.8686	1.5137	13.8588	1.4307	27.5509	1.5691
PostSUB	1.7476	0.5280	1.5225	0.4565	2.1249	0.7269	3.0727	1.0010
MS-DB	2.2173	0.3269	2.1018	0.1580	1.4978	0.1909	1.6799	0.1509
Thalamus	1.7093	0.4501	1.0427	0.2103	1.1849	0.2234	1.1212	0.2969
RSC	2.8141	0.6319	1.7410	0.3970	2.1658	0.4382	2.2247	0.6888
Vis cortex	1.9859	0.9604	4.1163	1.4331	7.6444	2.2371	2.2177	1.2674
Aud cortex	0.6553	0.3533	0.9941	0.4759	1.2035	0.2885	0.2911	0.1738
SS Cortex	0.0920	0.0435	0.0767	0.0383	0.0343	0.0122	0.0864	0.0531
TeA	0.9889	0.7338	0.4625	0.1840	0.5486	0.1165	0.2889	0.0871
Prh	0.6574	0.2115	0.2642	0.0875	0.4744	0.1725	0.5506	0.1024
Ect	0.3258	0.0775	0.1735	0.0408	0.3472	0.0884	0.4090	0.1204
LEC	0.7472	0.2130	0.8340	0.2597	0.4807	0.2291	1.0881	0.2779
MEC	1.4215	0.5267	0.9189	0.5871	0.8505	0.2668	2.0971	0.5510
# of mice	8		10		10		12	
# of starters	95	20	101	17	65	15	132	17
# of total labeled neurons	5,357	729	4,218	546	4,064	699	3,739	509
Overall connectivity	69	11	51	8	67	3	29	2

The data summary of CSI and PI values for WT young, WT middle-age, 5xFAD young, and 5xFAD middle-age mice. Data are presented as mean ± SEM.

Detailed statistical comparisons between the four groups of mice reveal significant CSI differences (Fig. 8, Table 4). Comparing age-matched WT and 5xFAD mice, the Vis cortex of the 5xFAD young group shows significantly stronger input connections to excitatory SUB neurons relative to the WT young group (Fig. 8*A*; Wilcoxon rank-sum test, *p* = 1.29 × 10^−2^). The CSI value of the WT young group is 1.275 ± 0.7136; the CSI value of the 5xFAD young group is 5.2217 ± 1.6207. For comparison between the two middle-age groups, 5xFAD middle-age mice show significantly smaller CSI values in the CA1_py, CA1_or, CA1_rad, MS-DB, Vis cortex, and Aud cortex compared with WT middle-age mice (Fig. 8*B*; Wilcoxon rank-sum test, *p* = 1.2 × 10^−4^, *p* = 4.46 × 10^−2^, *p* = 8.78 × 10^−3^, *p* = 3.33 × 10^−3^, *p* = 1.29 × 10^−2^, and *p* = 1.38 × 10^−2^, respectively). The CSI values of the WT middle-age group are 31.5 ± 4.3837 (CA1_py), 1.2242 ± 0.2206 (CA1_or), 0.2302 ± 0.0286 (CA1_rad), 1.0204 ± 0.1924 (MS-DB), 2.828 ± 1.5745 (Vis cortex), and 0.7486 ± 0.5241 (Aud cortex); the CSI values of the 5xFAD middle-age group are 13.8835 ± 1.3216 (CA1_py), 0.5687 ± 0.1026 (CA1_or), 0.1216 ± 0.0318 (CA1_rad), 0.4593 ± 0.0628 (MS-DB), 0.5301 ± 0.2877 (Vis cortex), and 0.0672 ± 0.0390 (Aud cortex), showing decreased connectivity in aged 5xFAD mice. In terms of comparison within the same genotype but at different ages, the WT young and WT middle-age groups show a significant difference in CSI values at the CA1_rad, SUB, and MS-DB (Fig. 8*C*; Wilcoxon rank-sum test, *p* = 8.78 × 10^−3^, *p* = 4.66 × 10^−2^, and *p* = 2.74 × 10^−2^, respectively). Among these regions, only the CA1_rad shows higher CSI values in WT middle-age mice. The CSI values of the WT young group are 0.116 ± 0.0193 (CA1_rad), 10.1783 ± 1.2574 (SUB), and 1.3175 ± 0.1816 (MS-DB); the CSI values of the WT middle-age group are 0.2302 ± 0.0286 (CA1_rad), 6.5282 ± 0.5425 (SUB), and 1.0204 ± 0.1924 (MS-DB). Age-related connectivity differences are more frequently observed in 5xFAD mice. For 5xFAD young and 5xFAD middle-age mice, the CA1_py, CA2_py, MS-DB, RSC, Vis cortex, Aud cortex, and TeA regions all show significantly weaker connections in 5xFAD middle-age relative to 5xFAD young mice (Fig. 8*D*; Wilcoxon rank-sum test, *p* = 1 × 10^−5^, *p* = 5.6 × 10^−4^, *p* = 4.03 × 10^−3^, *p* = 4.46 × 10^−2^, *p* = 1.2 × 10^−3^, *p* = 8.1 × 10^−4^, and *p* = 6.45 × 10^−3^, respectively). The CSI values of the 5xFAD young group are 40.7620 ± 2.3022 (CA1_py), 0.8725 ± 0.0868 (CA2_py), 0.9410 ± 0.1071 (MS-DB), 1.4492 ± 0.3497 (RSC), 5.2217 ± 1.6207 (Vis cortex), 0.8048 ± 0.2012 (Aud cortex), and 0.3566 ± 0.0686 (TeA); the CSI values of the 5xFAD middle-age group are 13.8835 ± 1.3216 (CA1_py), 0.4024 ± 0.0506 (CA2_py), 0.4593 ± 0.0628 (MS-DB), 0.6619 ± 0.2547 (RSC), 0.5301 ± 0.2877 (Vis cortex), 0.0672 ± 0.0390 (Aud cortex), and 0.0888 ± 0.0291 (TeA). To show the CSI values of specific impacted regions for all four mouse groups, example regions are plotted (Fig. 8*E*). Interestingly, the CSI value of the Vis cortex in the 5xFAD young group is much higher than in WT young and 5xFAD middle-age mice. Together, these data demonstrate significant alterations in presynaptic connectivity strength to SUB excitatory neurons in 5xFAD mice compared with WT mice. Moreover, these connectivity alterations show age-progressive features in WT and especially in 5xFAD mice.

**Table 4.** Statistical comparisons of CSI and PI values

	Genotype comparison				Age comparison			
	WT young versus 5xFAD young		WT middle versus 5xFAD middle		WT young versus WT middle		5xFAD young versus 5xFAD middle	
	*p* value	Significance	*p* value	Significance	*p* value	Significance	*p* value	Significance
CSI comparison								
CA1_py	0.35993		0.00012	***	0.31598		0.00001	****
CA1_or	0.36571		0.04461	*	0.57260		0.36571	
CA1_rad	0.40825		0.00878	**	0.00878	**	0.40825	
CA1_lmol	0.31424		0.31424		0.35993		0.31424	
CA2_py	0.76183		0.27076		0.27076		0.00056	***
CA2_or	0.45408		0.45408		0.57169		0.55594	
CA3_py	>0.999999		>0.999999		>0.999999		>0.999999	
CA3_or	0.44303		0.44303		0.96545		0.94545	
SUB	0.51479		0.46171		0.04662	*	0.46171	
PostSUB	>0.999999		>0.999999		>0.999999		>0.999999	
MS-DB	0.12199		0.00333	**	0.02736	*	0.00403	**
Thalamus	0.82856		0.24026		0.24026		0.06760	
RSC	0.57260		0.57260		0.16628		0.04461	*
Vis cortex	0.01289	*	0.01289	*	0.60088		0.00120	**
Aud cortex	0.18736		0.01379	*	0.39469		0.00081	***
SS cortex	0.66868		0.66868		0.66868		0.66868	
TeA	0.34562		0.45001		0.45976		0.00645	**
Prh	0.54433		0.58241		0.16084		0.16084	
Ect	0.69647		0.69647		0.17368		0.21440	
LEC	0.66668		0.71821		0.76183		0.71821	
MEC	0.58990		0.54856		0.54856		0.58990	
PI comparison								
CA1_py	0.27428		0.00120	**	0.19428		0.00120	**
CA1_or	0.36571		0.45615		0.36571		0.36571	
CA1_rad	0.35993		0.35993		0.01791	*	0.01791	*
CA1_lmol	0.31424		0.02754	*	0.89675		0.01760	*
CA2_py	>0.999999		>0.999999		>0.999999		>0.999999	
CA2_or	0.34334		0.09909		0.96545		0.83628	
CA3_py	>0.999999		0.96312		0.96312		0.96312	
CA3_or	0.21034		0.00601	**	0.21034		0.01791	*
SUB	0.13480		0.00007	****	0.23699		0.00005	****
PostSUB	0.82856		0.76347		0.76347		0.76347	
MS-DB	0.24582		0.24582		0.89675		0.60820	
Thalamus	0.71821		0.92287		0.71821		0.71821	
RSC	0.71986		0.82120		0.69126		0.82120	
Vis cortex	0.01934	*	0.05676		0.19323		0.01374	*
Aud cortex	0.18736		0.02664	*	0.30449		0.01324	*
SS cortex	0.86935		0.86935		0.86935		0.86935	
TeA	0.40614		0.54325		>0.999999		0.26917	
Prh	0.69647		0.05508		0.24398		0.37725	
Ect	>0.999999		0.58069		0.48796		>0.999999	
LEC	0.39160		0.66201		>0.999999		0.11689	
MEC	0.47463		0.06760		0.31598		0.16084	

The data summary of CSI and PI value group-wise comparisons. The comparisons include WT young versus 5xFAD young, WT middle-age versus 5xFAD middle-age, WT young versus WT middle-age, and 5xFAD young versus 5xFAD middle-age groups. Statistical significances are denoted by *p ≤ 0.05, **p ≤ 0.01, ***p ≤ 0.001, and ****p ≤ 0.0001. Statistical method: Wilcoxon rank-sum test. *P* values adjusted with the FDR Benjamini–Hochberg approach (desired FDR = 0.05).

### Quantitative analyses of regional-specific SUB input pattern changes in 5xFAD mice

**Figure 9.**
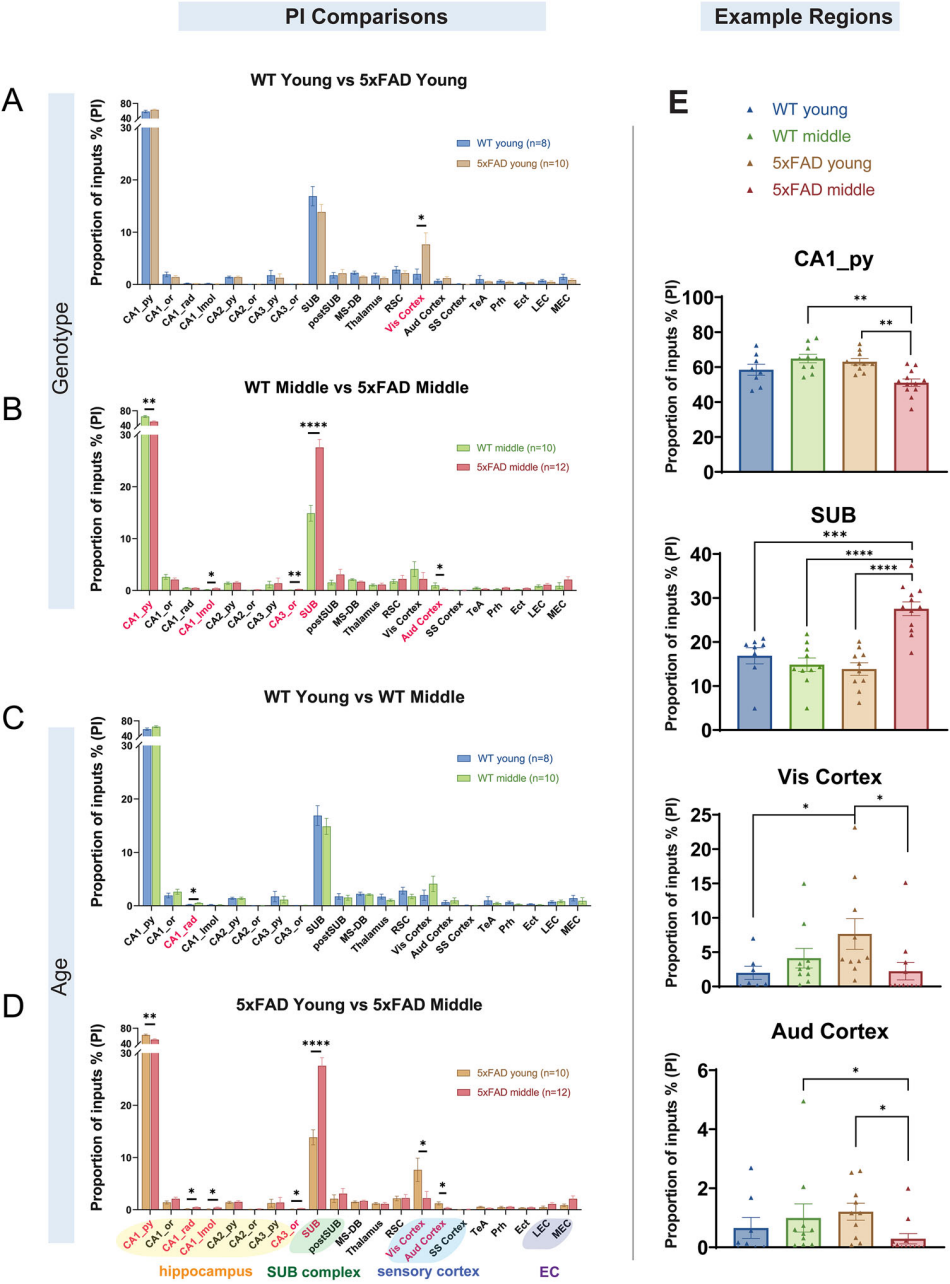
Quantification of the PI to SUB excitatory neurons. The PI is the ratio of the input neuron number in a subregion to the total input neuron number in a brain. The input regions include the hippocampal CA1, CA2, and CA3 sublayers, as well as the SUB, postSUB, thalamus, RSC, Vis cortex, Aud cortex, SS cortex, TeA, Prh, Ect, LEC, and MEC. The regions showing statistical differences are shown in red text. ***A***, 5xFAD young mice show significantly higher PI values than WT young mice in the Vis cortex (*p* = 1.93 × 10^−2^). ***B***, WT middle-age mice show significantly higher PI values than 5xFAD middle-age mice in the CA1_py and Aud cortex (*p* = 1.2 × 10^−3^ and *p* = 2.66 × 10^−2^, respectively). WT middle-age mice show significantly lower PI values than 5xFAD middle-age mice in the CA1_lmol, CA3_or, and SUB (*p* = 2.75 × 10^−2^, *p* = 6.01 × 10^−3^, and *p* = 7 × 10^−5^, respectively). ***C***, WT young mice show significantly lower PI values than WT middle-age mice in the CA1_rad (*p* = 1.79 × 10^−2^). ***D***, 5xFAD young mice show significantly higher PI values than 5xFAD middle-age mice in the CA1_py, Vis cortex, and Aud cortex (*p* = 1.2 × 10^−3^, *p* = 1.37 × 10^−2^, and *p* = 1.32 × 10^−2^, respectively). 5xFAD young mice show significantly lower PI values than 5xFAD middle-age mice in the CA1_rad, CA1_lmol, CA3_or, and SUB (*p* = 1.79 × 10^−2^, *p* = 1.76 × 10^−2^, *p* = 1.79 × 10^−2^, and *p* = 5.0 × 10^−5^, respectively). ***E***, PI comparisons of example regions across four groups of mice. Representative regions include the CA1_py, SUB, Vis cortex, and Aud cortex. The mouse numbers for each group are WT young, *n* = 8; WT middle-age, *n* = 10; 5xFAD young, *n* = 10; and 5xFAD middle-age, *n* = 12. The statistical test is the Wilcoxon rank-sum test. The *p* values are adjusted with the FDR Benjamini–Hochberg approach (desired FDR = 0.05). All data are represented with mean ± SEM. Statistical significances are denoted by *p ≤ 0.05, **p ≤ 0.01, ***p ≤ 0.001, and ****p ≤ 0.0001. See also Tables 3 and 4.

To investigate whether the SUB input connectivity patterns shift in 5xFAD mice with their significantly altered connectivity, we measured the PI index based on the proportion of each input region for the total inputs (Fig. 9). The PI index is represented by the fraction of presynaptic neurons in each input region over the entire brain inputs and provides a more global measure of connectivity contribution for a given region. For comparisons between WT young and 5xFAD young mice, the 5xFAD young mice show significantly higher PI values in the Vis cortex (Fig. 9*A*; *p* = 1.93 × 10^−2^). The PI values of WT young and 5xFAD young mice are 1.9859 ± 0.9604 and 7.6444 ± 2.2371, respectively. For WT middle-age and 5xFAD middle-age mice, 5xFAD middle-age mice show significantly lower PI values in the CA1_py and Aud cortex (*p* = 1.2 × 10^−3^ and *p* = 2.66 × 10^−2^, respectively) and higher PI values in the CA1_lmol, CA3_or, and SUB (*p* = 2.75 × 10^−2^, *p* = 6.01 × 10^−3^, and *p* = 7 × 10^−5^ respectively; Fig. 9*B*). The PI values for WT middle-age are 64.8701 ± 2.4048 (CA1_py), 0.9941 ± 0.4759 (Aud cortex), 0.1572 ± 0.0393 (CA1_lmol), 0.0992 ± 0.0186 (CA3_or), and 14.8686 ± 1.5137 (SUB). The PI values for 5xFAD middle-age are 51.0676 ± 2.1452 (CA1_py), 0.2911 ± 0.1738 (Aud cortex), 0.4002 ± 0.1152 (CA1_lmol), 0.2486 ± 0.0324 (CA3_or), and 27.5509 ± 1.5691 (SUB). For comparisons between ages, WT young mice show lower PI values in the CA1_rad than WT middle-age mice (Fig. 9*C*; *p* = 1.79 × 10^−2^). The PI values of WT young and WT middle-age mice are 0.2115 ± 0.0556, and 0.5114 ± 0.0625, respectively. Compared to 5xFAD young mice, 5xFAD middle-age mice show significantly lower connections in the CA1_py, Vis cortex, and Aud cortex (*p* = 1.2 × 10^−3^, *p* = 1.37 × 10^−2^, and *p* = 1.32 × 10^−2^, respectively) and higher PI values in the CA1_rad, CA1_lmol, CA3_or, and SUB (*p* = 1.79 × 10^−2^, *p* = 1.76 × 10^−2^, *p* = 1.79 × 10^−2^, and *p* = 5.0 × 10^−5^, respectively; Fig. 9*D*). The PI values of the 5xFAD young group are 63.0325 ± 1.8142 (CA1_py), 7.6444 ± 2.2371 (Vis cortex), 1.2035 ± 0.2885 (Aud cortex), 0.1582 ± 0.0507 (CA1_rad), 0.1127 ± 0.0514 (CA1_lmol), 0.1258 ± 0.0258 (CA3_or), and 13.8588 ± 1.4307 (SUB). The PI values of the 5xFAD middle-age group are 51.0676 ± 2.1452 (CA1_py), 2.2177 ± 1.2674 (Vis cortex), 0.2911 ± 0.1738 (Aud cortex), 0.4526 ± 0.0978 (CA1_rad), 0.4002 ± 0.1152 (CA1_lmol), 0.2486 ± 0.0324 (CA3_or), and 27.5509 ± 1.5691 (SUB). To show the PI values of specific impacted regions for all four mouse groups, example regions are plotted (Fig. 9*E*). Notably, the SUB PI of the 5xFAD middle-age group is higher than that of the other three mouse groups. Together, these data support the idea that compensatory circuit input changes may occur in response to connectivity losses in AD conditions.

### Sex-specific differences in SUB input connectivity strengths and patterns

**Figure 10.**
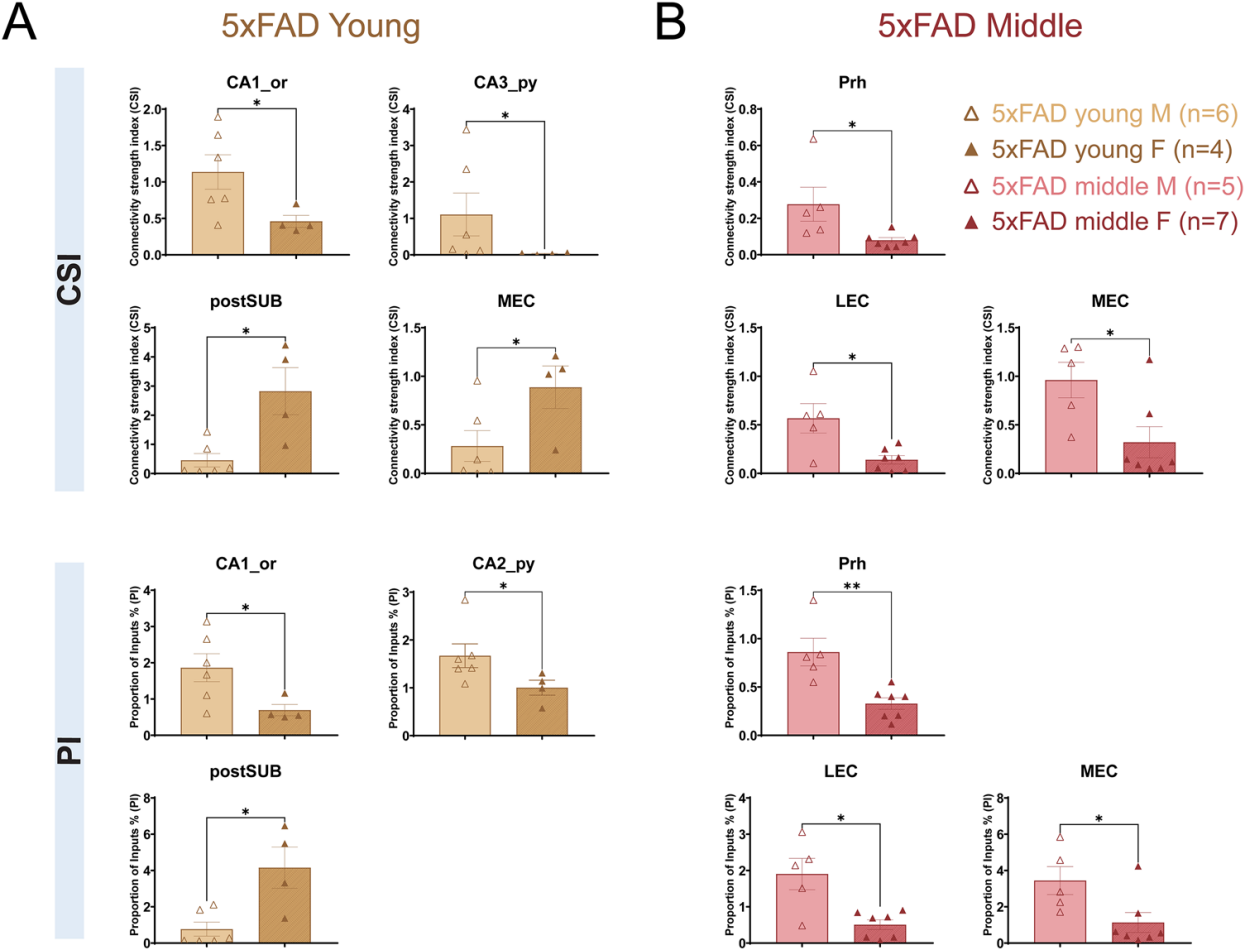
Sex differences of CSI and PI measurements in 5xFAD young and middle-age mice. ***A***, Sex differences of the 5xFAD young group. The CSI differences are observed for the CA1_or, CA3_py, postSUB, and MEC (Wilcoxon rank-sum test, *p* = 1.9 × 10^−2^, *p* = 3.81 × 10^−2^, *p* = 1.9 × 10^−2^, and *p* = 3.81 × 10^−2^, respectively). The PI differences are observed for the CA1_or, CA2_py, and postSUB (Wilcoxon rank-sum test, *p* = 3.81 × 10^−2^, *p* = 3.81 × 10^−2^, and *p* = 3.81 × 10^−2^, respectively). ***B***, Sex differences of the 5xFAD middle-age group. The CSI differences are observed for the Prh, LEC, and MEC (Wilcoxon rank-sum test, *p* = 1.01 × 10^−2^, *p* = 3.03 × 10^−2^, and *p* = 3.03 × 10^−2^, respectively). The PI differences are observed in the Prh, LEC, and MEC (Wilcoxon rank-sum test, *p* = 5.1 × 10^−3^, *p* = 3.03 × 10^−2^, and *p* = 1.77 × 10^−2^, respectively). The mouse numbers for each group are 5xFAD young (male, *n* = 4; female, *n* = 6) and 5xFAD middle-age (male, *n* = 5; female, *n* = 7). The statistical test is the Wilcoxon rank-sum test. All data are represented with mean ± SEM. Statistical significances are denoted by *p ≤ 0.05, **p ≤ 0.01, ***p ≤ 0.001, and ****p ≤ 0.0001.

In human patients, AD is more common in females than males on an age-matched basis (Seshadri et al., 1997). We asked whether sex differences in SUB neuron connectivity strengths and patterns may be recapitulated in the 5xFAD mouse model. The CSI and PI values were compared between male and female mice for each group pair (Fig. 10, Tables 5, 6). Significant sex differences are observed in all four groups of mice when comparing CSI and PI values. Specifically, for the WT young group, male mice show higher CSI values than female mice for MS-DB (*p* = 3.57 × 10^−2^) and lower PI values in the postSUB (*p* = 3.57 × 10^−2^; Table 6). For the WT middle-age group, male mice show higher CSI values in the CA2_or, CA3_or, and SUB (*p* = 3.17 × 10^−2^, *p* = 7.9 × 10^−3^, and *p* = 3.17 × 10^−2^, respectively). WT middle-age male mice show higher PI values than females in the CA3_or and SUB (*p* = 3.17 × 10^−2^ and *p* = 3.17 × 10^−2^, respectively; Table 6). For 5xFAD mice, there are more sex differences (Fig. 10). For the 5xFAD young group, male mice show higher CSI values in the CA1_or and CA3_py (*p* = 1.9 × 10^−2^ and *p* = 3.81 × 10^−2^, respectively) and lower CSI values in the postSUB and MEC (*p* = 1.9 × 10^−2^ and *p* = 3.81 × 10^−2^, respectively). 5xFAD young male mice show higher PI values in the CA1_or and CA2_py and lower CSI values in the postSUB than female mice (*p* = 3.81 × 10^−2^, *p* = 3.81 × 10^−2^, and *p* = 3.81 × 10^−2^, respectively). For the 5xFAD middle-age group, male mice show higher CSI values than female mice in the Prh, LEC, and MEC (*p* = 1.01 × 10^−2^, *p* = 3.03 × 10^−2^, and *p* = 3.03 × 10^−2^, respectively). For PI values, 5xFAD middle-age male mice show higher PI values than female mice in the Prh, LEC, and MEC (*p* = 5.1 × 10^−3^, *p* = 3.03 × 10^−2^, and *p* = 1.77 × 10^−2^, respectively). Overall, there are significant sex differences in multiple brain regions, and this sex difference is more prominent in 5xFAD mice than in WT mice for both ages. These results indicate that the circuit connections to SUB excitatory neurons from different brain regions may be differentially impacted by sex-related factors, especially in 5xFAD mice.

**Table 5.** Data summary of sex-specific CSI and PI measurements

	WT young male		WT young female		WT middle male		WT middle female		5xFAD young male		5xFAD young female		5xFAD middle male		5xFAD middle female	
	Mean	SEM	Mean	SEM	Mean	SEM	Mean	SEM	Mean	SEM	Mean	SEM	Mean	SEM	Mean	SEM
CSI summary																
CA1_py	42.9091	12.2310	34.7270	5.2214	29.0351	5.1714	33.9649	7.5297	40.2835	3.2403	41.4797	3.6353	15.2042	2.3854	12.9402	1.5570
CA1_or	1.7879	0.8642	0.8493	0.1457	1.0370	0.2123	1.4113	0.3954	1.1376	0.2363	0.4597	0.0822	0.5438	0.1110	0.5866	0.1645
CA1_rad	0.1510	0.0211	0.0950	0.0247	0.2026	0.0374	0.2579	0.0434	0.1342	0.0473	0.0449	0.0150	0.0852	0.0161	0.1477	0.0528
CA1_lmol	0.1911	0.0949	0.0603	0.0142	0.0745	0.0211	0.0510	0.0215	0.0657	0.0411	0.0595	0.0188	0.0877	0.0203	0.1128	0.0440
CA2_py	0.9353	0.1983	0.7653	0.1075	0.7019	0.1284	0.7734	0.4535	1.0114	0.1015	0.6642	0.0812	0.3530	0.0887	0.4377	0.0617
CA2_or	0.0181	0.0090	0.0366	0.0136	0.0384	0.0102	0.0084	0.0045	0.0559	0.0182	0.0618	0.0195	0.0421	0.0202	0.0450	0.0183
CA3_py	2.4608	1.2414	0.1374	0.0819	0.3693	0.1916	0.4161	0.3432	1.1074	0.5873	0.0296	0.0124	0.1569	0.0470	0.4800	0.3989
CA3_or	0.0738	0.0251	0.0317	0.0102	0.0577	0.0027	0.0250	0.0104	0.0848	0.0141	0.0599	0.0265	0.0632	0.0190	0.0724	0.0147
SUB	10.6596	2.6834	9.8896	1.4870	7.7774	0.4238	5.2789	0.6037	7.6731	1.2193	10.5810	0.9464	8.2400	1.3706	6.9236	0.8505
PostSUB	0.4306	0.0779	1.2640	0.3240	0.4970	0.2371	1.3884	0.8776	0.4543	0.2307	2.8238	0.8060	0.8778	0.1983	0.9990	0.6549
MS-DB	1.8243	0.3018	1.0134	0.0583	0.9049	0.0536	1.1360	0.3963	0.9502	0.1318	0.9271	0.2060	0.4702	0.0992	0.4516	0.0877
Thalamus	0.4501	0.1973	1.1735	0.3278	0.5409	0.1744	0.3896	0.0758	0.5719	0.1623	1.0193	0.1178	0.3824	0.0413	0.3018	0.1921
RSC	1.7087	0.9882	1.8514	0.5427	0.7237	0.2652	1.2522	0.7637	0.9900	0.2688	2.1380	0.6878	0.6291	0.0445	0.6853	0.4503
Vis cortex	2.6864	2.6407	0.7104	0.3050	0.7550	0.2404	4.9011	2.9914	5.5455	2.4395	4.7361	2.1658	0.0374	0.0136	0.8820	0.4588
Aud cortex	1.6675	1.0918	0.0970	0.0580	0.1509	0.0642	1.3464	1.0264	0.7917	0.2753	0.8244	0.3376	0.0025	0.0016	0.1134	0.0626
SS cortex	0.1274	0.0787	0.0318	0.0178	0.0186	0.0080	0.0964	0.0835	0.0219	0.0123	0.0223	0.0085	0.0035	0.0015	0.0360	0.0200
TeA	2.8949	2.7756	0.1613	0.0565	0.0787	0.0336	0.5314	0.2831	0.3616	0.1036	0.3490	0.0919	0.0860	0.0532	0.0908	0.0362
Prh	0.7119	0.3397	0.2246	0.0810	0.1132	0.0169	0.1044	0.0515	0.2847	0.1224	0.2939	0.1397	0.2772	0.0937	0.0797	0.0145
Ect	0.4496	0.2008	0.1333	0.0572	0.0792	0.0275	0.0934	0.0496	0.2207	0.0981	0.2595	0.1004	0.2067	0.1016	0.0718	0.0295
LEC	0.6528	0.3537	0.3170	0.1063	0.4933	0.1562	0.2037	0.0447	0.2568	0.1801	0.2946	0.0762	0.5671	0.1518	0.1399	0.0435
MEC	0.5968	0.2774	0.8982	0.4260	0.6872	0.4344	0.1312	0.0821	0.2812	0.1586	0.8867	0.2191	0.9609	0.1825	0.3201	0.1603
PI summary																
CA1_py	50.5757	3.2526	63.2287	3.1864	64.0740	3.4701	65.6662	3.6968	64.5390	2.7946	60.7729	1.5243	52.1118	2.6882	50.3218	3.2863
CA1_or	2.3875	1.1582	1.6367	0.3367	2.4741	0.6328	2.7875	0.7527	1.8629	0.3860	0.6946	0.1556	1.8186	0.2451	2.2809	0.5424
CA1_rad	0.2274	0.0908	0.2019	0.0783	0.4928	0.1191	0.5299	0.0565	0.2186	0.0754	0.0676	0.0226	0.3011	0.0575	0.5608	0.1543
CA1_lmol	0.2800	0.1309	0.1323	0.0466	0.1662	0.0338	0.1482	0.0760	0.1252	0.0860	0.0938	0.0330	0.2983	0.0621	0.4730	0.1943
CA2_py	1.4045	0.5303	1.4050	0.0804	1.5931	0.2552	1.2204	0.3758	1.6700	0.2483	1.0028	0.1566	1.1511	0.1526	1.7750	0.3148
CA2_or	0.0310	0.0165	0.0643	0.0183	0.0919	0.0244	0.0231	0.0113	0.0982	0.0328	0.0976	0.0345	0.1229	0.0385	0.1934	0.0859
CA3_py	4.0921	2.0561	0.3262	0.2263	0.9925	0.5877	1.3077	1.1849	2.0701	1.1955	0.0407	0.0156	0.4929	0.0803	2.0228	1.7423
CA3_or	0.0823	0.0157	0.0652	0.0228	0.1360	0.0123	0.0625	0.0269	0.1454	0.0319	0.0963	0.0441	0.1974	0.0311	0.2853	0.0480
SUB	15.2455	5.1588	17.8589	0.9671	18.1437	1.3884	11.5936	1.7380	12.4974	1.8416	15.9009	2.1347	27.9713	1.6107	27.2506	2.5429
PostSUB	0.5361	0.0678	2.4744	0.6535	1.1785	0.6207	1.8664	0.7025	0.7697	0.3836	4.1576	1.1400	3.0415	0.5101	3.0950	1.7393
MS-DB	2.5588	0.7617	2.0124	0.3117	2.1252	0.2090	2.0784	0.2616	1.5778	0.2631	1.3778	0.3040	1.5772	0.0958	1.7532	0.2551
Thalamus	0.7584	0.3889	2.2798	0.5522	1.2775	0.3898	0.8079	0.1395	0.9689	0.3375	1.5089	0.1772	1.3694	0.1612	0.9439	0.5020
RSC	1.6603	0.4979	3.5065	0.8510	1.7324	0.6405	1.7496	0.5468	1.5832	0.3905	3.0396	0.7965	2.3727	0.4423	2.1190	1.1805
Vis cortex	3.5469	9.5324	1.3614	0.6376	1.6289	0.4941	6.6038	2.4299	8.1577	3.3488	6.8743	3.0282	0.1208	0.0333	3.7154	2.0374
Aud cortex	1.4570	0.7845	0.1743	0.1026	0.3035	0.1028	1.6846	0.8777	1.2092	0.3982	1.1950	0.4772	0.0066	0.0040	0.4943	0.2800
SS cortex	0.1454	0.1078	0.0599	0.0334	0.0426	0.0202	0.1109	0.0749	0.0354	0.0195	0.0325	0.0129	0.0109	0.0046	0.1403	0.0878
TeA	2.1409	1.9823	0.2976	0.1020	0.1580	0.0598	0.7670	0.3200	0.5785	0.1838	0.5038	0.1275	0.2467	0.1613	0.3191	0.1053
Prh	1.0440	0.4758	0.4255	0.1465	0.2664	0.0435	0.2620	0.1804	0.5215	0.2787	0.4038	0.1621	0.8612	0.1434	0.3288	0.0591
Ect	0.4609	0.1409	0.2447	0.0804	0.1975	0.0720	0.1494	0.0450	0.3386	0.1355	0.3601	0.1123	0.5803	0.2180	0.2866	0.1301
LEC	0.9827	0.4826	0.6059	0.2094	1.2550	0.4542	0.4130	0.0931	0.5077	0.3894	0.4404	0.1166	1.9025	0.4318	0.5063	0.1363
MEC	1.0603	0.6549	1.6383	0.7854	1.6699	1.1236	0.1678	0.0815	0.5249	0.3304	1.3390	0.3586	3.4448	0.7696	1.1345	0.5491
# of mice	3		5		5		5		6		4		5		7	
# of starters	93	40	96	25	120	22	83	26	73	26	54	4	183	15	95	14
# of total labeled neurons	6,173	1,835	4,867	564	5,101	612	3,336	759	4,261	1,187	3,771	299	5,366	411	2,577	438
Overall connectivity	90	27	57	7	46	5	55	15	64	4	70	5	31	5	27	3

The data summary for sex-specific CSI and PI values of WT young, WT middle-age, 5xFAD young, and 5xFAD middle-age mice. Data are presented as mean ± SEM.

**Table 6.** Statistical comparisons of CSI and PI values between sexes

	WT young male versus WT young female		WT middle male versus WT middle female		5xFAD young male versus 5xFAD young female		5xFAD middle male versus 5xFAD middle female	
	*p* value	Significance	*p* value	Significance	*p* value	Significance	*p* value	Significance
CSI comparison								
CA1_py	0.7857		0.8413		>0.9999		0.4318	
CA1_or	0.3929		0.5476		0.0190	*	0.8763	
CA1_rad	0.2500		0.8413		0.2571		0.6755	
CA1_lmol	0.2500		0.3095		0.6095		0.8763	
CA2_py	0.2500		0.3095		0.0667		0.2677	
CA2_or	0.7857		0.0317	*	0.7095		0.6389	
CA3_py	0.1250		>0.9999		0.0381	*	0.4318	
CA3_or	0.2500		0.0079	**	0.5714		0.6389	
SUB	>0.9999		0.0317	*	0.1143		0.5030	
PostSUB	0.0714		0.4206		0.0190	*	0.2020	
MS-DB	0.0357	*	0.3095		0.9143		0.8763	
Thalamus	0.1429		>0.9999		0.1143		0.0732	
RSC	0.5714		0.6905		0.1143		0.1490	
Vis cortex	>0.9999		0.2222		0.7619		0.4318	
Aud cortex	0.4464		0.0952		0.9413		0.0922	
SS cortex	0.3750		>0.9999		0.8000		0.1263	
TeA	>0.9999		0.4206		0.9143		0.6755	
Prh	0.2500		0.4206		0.7619		0.0101	*
Ect	0.2500		>0.9999		0.7619		0.2020	
LEC	0.5714		0.2222		0.3238		0.0303	*
MEC	>0.9999		0.2222		0.0381	*	0.0303	*
PI comparison								
CA1_py	0.0714		>0.9999		0.4762		0.8763	
CA1_or	>0.9999		0.8413		0.0381	*	0.8763	
CA1_rad	0.7857		0.6905		0.2571		0.4318	
CA1_lmol	0.3929		0.4206		0.6095		0.7551	
CA2_py	0.5714		0.5476		0.0381	*	0.2020	
CA2_or	0.3929		0.0556		>0.9999		0.6389	
CA3_py	0.2321		>0.9999		0.0667		0.3434	
CA3_or	>0.9999		0.0317	*	0.3524		0.3434	
SUB	0.5714		0.0317	*	0.2571		>0.9999	
PostSUB	0.0357	*	0.4206		0.0381	*	0.2677	
MS-DB	0.5714		>0.9999		>0.9999		0.4318	
Thalamus	0.1429		0.6905		0.2571		0.1490	
RSC	0.3929		0.5476		0.1143		0.2677	
Vis cortex	>0.9999		0.0556		>0.9999		0.4318	
Aud cortex	0.4464		0.0952		>0.9999		0.0922	
SS cortex	0.7857		>0.9999		0.8000		0.1263	
TeA	>0.9999		0.2222		0.9143		0.6755	
Prh	0.3929		0.1508		0.9143		0.0051	**
Ect	0.2500		0.6905		0.7619		0.3434	
LEC	0.5714		0.1508		0.2381		0.0303	*
MEC	>0.9999		0.2222		0.1714		0.0177	*

The data summary of CSI and PI value comparisons between sexes for WT young, middle-age, 5xFAD young, and 5xFAD middle-age mice. Statistical significances are denoted by *p ≤ 0.05, **p ≤ 0.01, ***p ≤ 0.001, and ****p ≤ 0.0001. Statistical method: Wilcoxon rank-sum test.

## Discussion

While the past work focused on neuropathological features, AD is increasingly considered as a neural circuit disorder. In the work described above, we applied the monosynaptic rabies tracing approach to investigate alterations in neural circuit connectivity of SUB excitatory neurons in age-matched, gender-balanced WT, and 5xFAD mice. Our immunostaining results of the SUB region are in strong agreement with 5xFAD neuropathology data reported in the previously published studies (Oakley et al., 2006; Jawhar et al., 2012). In parallel to our immunostaining results that show age-progressive amyloid deposition of the SUB in 5xFAD mice, our monosynaptic rabies virus tracing reveals pathological impairments of excitatory SUB input connectivity. We mapped and compared local and long-range presynaptic inputs to SUB excitatory neurons from multiple brain regions in age-matched WT and 5xFAD mouse groups at young and middle ages. Quantification of the age-matched WT and 5xFAD SUB neural tracing reveals age- and genotype-dependent alterations. Our results also demonstrate prominent sex differences in AD-induced circuit connectivity alterations.

Our monosynaptic rabies tracing method is robust, as it works through a highly specific interaction between the TVA receptor and EnvA-pseudotyped rabies. To ensure the precise labeling of the dorsal SUB, we used a small amount of helper AAV (0.05 μl) to prevent leaky labeling of starter cells outside the SUB, followed by a typical amount of 0.4 μl of the EnvA-pseudotyped RV injection, which was established in the previous protocols (Lin et al., 2021; Ye et al., 2022). Moreover, cortical rabies labeling indeed reflects the presynaptic input labeling of SUB starter neurons, instead of leaky labeling. This is because of the technical nature of our monosynaptic rabies tracing approach. In our SUB experiments, only the starter cells in the SUB can take up EnvA-ΔG rabies virus, as they have the required TVA receptor expression. However, the cortical cells and their axons do not express TVA; thus, technically, they cannot be directly infected and labeled by the EnvA-pseudotyped rabies virus. This is verified by our control experiments of single injections of pseudotyped rabies virus alone that did not yield artifactual labeling. In our previously published studies and others investigating hippocampal CA1 circuitry using monosynaptic rabies tracing, no rabies labeling was observed in the cortex above the CA1 injection site, arguing against the possibility that pseudotyped rabies virus infects through the damaged axons and other processes that would bypass the EnvA-mediated specificity (Sun et al., 2014; Tao et al., 2021; Ye et al., 2022). Overall, the presynaptic input regions identified by this study and the CSI values of the WT young group are generally concordant with an earlier SUB rabies tracing study using a CaMK2a-Cre: TVA mouse line (Sun et al., 2019), with the strongest input from the hippocampal CA1 and significant inputs from the EC, Vis, Aud, and other regions. We observed robust labeling from the Vis cortex and SS cortex. These observations are supported by the Allen Brain Connectivity and Janelia MouseLight datasets (Oh et al., 2014; Winnubst et al., 2019), where AAV injections from the Vis cortex and SS cortex result in axon labeling in the SUB region. Note that we do not anticipate that the rabies tracing method labels every input to each neuron with 100% efficiency. Nevertheless, the rabies virus tracing technique used in this study holds significant promise as a powerful tool for quantitatively assessing connectivity when comparing between WT and AD model mice.

In the current study, we identify substantial noncanonical inputs from the hippocampal CA3, which has not been reported previously. Moreover, this study provides the first viral genetic-based anatomical evidence verifying that SUB neurons receive strong recurrent connections, supporting earlier anatomy and whole-cell recording studies (Harris et al., 2001; Bohm et al., 2015). Our Cre-dependent viral strategy facilitates precise mapping of the local SUB circuitry because this molecular tool restricts the expression of optimized rabies glycoprotein (oG) and the highly specific TVA receptor (TC66T) exclusively within CaMKII + SUB neurons. Therefore, the presynaptic-labeled SUB neurons are indeed local input neurons, eliminating any artifactual possibility of leakage. Furthermore, our results demonstrate that the local intrinsic connections to SUB excitatory neurons are resistant in AD, as the CSI values of the WT and 5xFAD groups at both ages are consistent. However, the proportional input of SUB local connection in the AD middle-age group is significantly higher due to the decrease of other input regions, indicating a potential compensatory effect of AD on SUB connections.

It is noteworthy that the visual cortical inputs to SUB show a significantly increased connectivity in young 5xFAD mice compared with WT mice, followed by a decreased connectivity in middle-age 5xFAD mice. It is increasingly recognized that early stages of AD are characterized by neuronal hyperexcitability, followed by a transition toward hypoexcitability at later ages. Therefore, the increased connectivity of the Vis cortex to the SUB in young AD mice may be attributed to enhanced synaptic connection strengths due to hyperexcitability in the initial phases of the disease, as an earlier study shows that rabies labeling may be activity-dependent (Beier et al., 2017). The Vis cortex is generally considered an understudied brain region in AD. However, recently increasing evidence suggests that the Vis cortex may be functionally implicated in AD. It has been shown that 40 Hz gamma frequency entrainment attenuates amyloid load in the Vis cortex and hippocampal CA1 of young 5xFAD mice, reduces neuron and spine loss, and improves behavioral performance (Iaccarino et al., 2016). This indicates a potential functional network between the Vis cortex and the hippocampal formation system. Indeed, a previous study shows that the projection from the Vis cortex to the SUB is implicated in object location memory (Sun et al., 2019). 5xFAD mice display impairment of object location memory at 8–10 months but not 4–5 months old (Zhang et al., 2023). This implies that the impairment of the Vis cortex inputs to the SUB could be involved in spatial and object-related learning and memory deficits.

Importantly, our viral tracing results demonstrate prominent sex differences in circuit alterations in 5xFAD mice. In human patients, the risk of women having AD is higher than in men due to a series of factors such as longevity, hormonal level, genetics, inflammation, and other sociocultural factors (Nebel et al., 2018; Zhu et al., 2021). While the underlying mechanism of the sex difference remains elusive, there is evidence showing that the association between AD pathology and clinical AD was substantially stronger in women than in men (Barnes et al., 2005). Moreover, a study has shown that women had higher CSF total tau and Aβ42 levels, more rapid cognitive decline, and hippocampal atrophy, indicating that they experience worse pathologic alterations than men (Koran et al., 2017). Differences between sexes were also revealed by transcriptomic analysis of AD pathology-associated marker genes, and females show higher expression (Mathys et al., 2019). In this study, the amyloid quantification shows that female mice exhibit more severe amyloid plaques and intraneuronal accumulation in both young and middle-age 5xFAD mice. Importantly, there are notable disparities in neural circuit connectivity to the SUB between the sexes. The sex-specific impacted regions are primarily the hippocampal subregions, postSUB, Prh, and EC: all of these areas are critical for learning and memory. Interestingly, in young 5xFAD mice, female mice show higher connectivity than male mice in certain regions. However, in middle-age 5xFAD mice, female mice all show weaker connectivity compared with male mice for regions with sex differences. This further implies sex-specific differences in the timing of hyperexcitability in young 5xFAD mice.

Together, our research shows significant connectivity strength alterations and connectivity pattern shifts in AD model mice. These new findings may provide valuable insights into addressing AD neural mechanisms as a neural circuit disorder. The sex-specific differences are interesting and may offer a new perspective to tackle this disease. Future neural circuit studies could pave the way for improved therapeutic interventions that may slow down or mitigate this disease during its early stages in AD patients.
